# Computational analysis, Urbach energy and Judd–Ofelt parameter of warm Sm^3+^ complexes having applications in photovoltaic and display devices†

**DOI:** 10.1039/d2ra05796d

**Published:** 2022-12-15

**Authors:** Poonam Kumari, Vaishnavi Lather, Savita Khatri, Pratibha Ahlawat, Harkesh Sehrawat, S. P. Khatkar, V. B. Taxak, Rajesh Kumar

**Affiliations:** University Institute of Engineering and Technology, Maharshi Dayanand University Rohtak 124001 India lather_rajesh@yahoo.com +91 9034070027; Department of Chemistry, Maharshi Dayanand University Rohtak 124001 India; Shri Guru Ram Rai Institute of Medical and Health Sciences Dehradun 248001 India

## Abstract

In this work, six reddish orange Sm^3+^ complexes were synthesized using organic ligand (L) and secondary ligands having hetero atoms by a one-step significant liquid-assisted grinding method and were characterized by spectroscopic techniques. The Urbach energy and band gap energy of the complexes were inspected by a linear fit. Using a least square fitting method, the Judd–Ofelt parameter and radiative properties were also determined. Thermal analysis, colorimetric analysis, luminescence decay time and anti-microbial properties of complexes were studied. The luminescence emission spectra of binary and ternary complexes displayed three characteristic peaks at 565, 603 and 650 nm in the powder form and four peaks at 563, 605, 646 and 703 nm in a solution phase due to ^4^G_5/2_ → ^6^H_5/2,_^4^G_5/2_ → ^6^H_7/2,_^4^G_5/2_ → ^6^H_9/2_ and ^4^G_5/2_ → ^6^H_11/2_ transitions respectively. The most intense transition in the solid phase (^4^G_5/2_ → ^6^H_7/2_) is accountable for orange color, and in the solution form, the highly luminescent peak (^4^G_5/2_ → ^6^H_9/2_) is responsible for reddish orange color of Sm^3+^ complexes. PXRD and SEM analyses suggested that the complexes possess a nanoparticle grain size with crystalline nature. The decent optoelectrical properties of title complexes in the orangish-red visible domain indicated possible applications in the manufacturing of display and optoelectronic devices.

## Abstract 2

Six reddish orange Sm^3+^ complexes were synthesized with the help of organic ligand and secondary ligands *via* one-step significant liquid-assisted grinding method and characterized spectroscopically.

## Graphical abstract

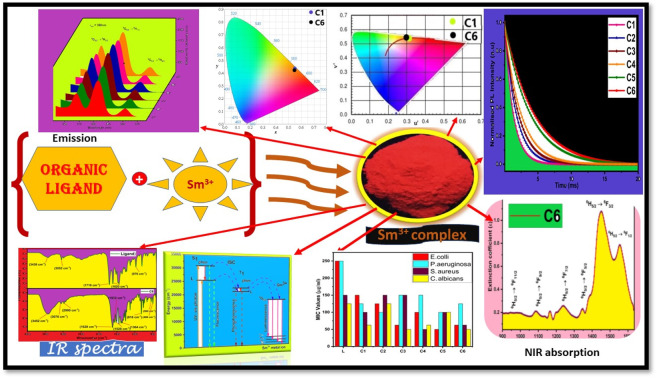

## 1 Introduction

The present scenario of technical evaluation in the field of global level illumination is promptly surging for intriguing material and science innovators to achieve present illumination targets by exploring energy saving display devices in an environmentally benign manner.^1^ Thus, organic lanthanide complexes mark a major achievement to complete ecological illumination requirements and are also eco-friendly for the environment with respect to incandescent and fluorescent lamps.^2–4^ Recent research focuses on the synthesis of highly energy conserving organic lanthanide complexes by a liquid-assisted grounding method,^5^ which indicated their successful use in display devices, optoelectronic appliances, bioimaging, solar cells, *etc.*^6–10^ These complexes have distinct characteristics such as large Stokes shift, line-like emission bands, higher life time and high quantum yield.^11,12^

To synthesize organic lanthanide complexes, two dominant sources are essential: one is an organic moiety and the other is a selected lanthanide metal ion. Much work is devoted to the synthesis of Eu^3+^ and Tb^3+^ complexes, which are characterized by brilliant luminescence and better emission in the red and green regions respectively.^13–15^ In comparison with Eu^3+^ and Tb^3+^ ions, the optical properties of Sm^3+^ ions are less studied due to a smaller energy gap between the resonating level (^4^G_5/2_) and the subsequent energy state.^5,9,16,17^ However, the Sm^3+^ ion has some other extraordinary properties such as dual emitting behaviour in UV-visible as well as NIR regions and also emission of various colors (red, orange and green) in a single complex that attract the attention of authors.^18–20^ However, the 4f–4f transition of lanthanide(iii) ions is Laporte forbidden, which results in a low emission intensity.^21,22^ To overpower lesser intensity and for the effective transfer of energy, organic ligands with a high absorption coefficient, a bidentate donor site and an extensive π conjugation system are required for chelation with lanthanide(iii) ions. The impact of chelation of ligands on the optical characteristics of lanthanide(iii) ions is known as the “antenna effect”.^23–25^ Ligands have a higher affinity to coordinate with lanthanide(iii) ions *via* oxygen (O) and nitrogen (N) atom donor sites. Generally, β-diketone, keto carboxylic acid, aromatic carboxylic acid and chromones are in use to sensitize lanthanide(iii) ions and also to increase their emission intensity. Among all the organic ligands, authors selected 7-methyl-4-oxo-1-(1,1,2,2,2-pentadeuterioethyl)-1,8-naphthyridine-3-carboxylic acid (L) as a primary ligand and neocuproine (neo), bathophenanthroline (batho), 2,2′-bipyridyl (bipy), 1,10-phenanthroline (phen) and 5-6-dimethyl-1,10-phenanthroline (dmph) as secondary ligands. Secondary ligands not only support the main ligand in enhancing the emission intensity but also reduce the quenching of illumination by substituting the water molecule. The Judd–Ofelt theory is an aspect to determine the symmetry, rigidity and covalence of lanthanide(iii) complexes. Furthermore, selecting an appropriate organic ligand and secondary ligands contribute to the preparation of proficient optical materials.

In the present work, the selected organic ligand is a quinolone and utilising secondary ligands, six Sm^3+^ complexes, namely [Sm(L)_3_·2H_2_O]·3H_2_O (C1), [Sm(L)_3_·bypy]·3H_2_O (C2), [Sm(L)_3_·dmph]·3H_2_O (C3), [Sm(L)_3_·batho]·3H_2_O (C4), [Sm(L)_3_·neo]·3H_2_O (C5) and [Sm(L)_3_·phen]·3H_2_O (C6), were synthesized by an environmentally benign solvent-assisted grinding method. The liquid-assisted grinding method avoided the drawback of the solution-based approach, especially solubility issues.^26^ To assess the optoelectrical properties of organic Sm^3+^ complexes, the band gap energy (*E*_g_) value and Urbach energy were computed using the reflectance electronic spectra.^27,28^ Extensive research has been done in the pharmaceutical field of lanthanide complexes with quinolones as ligands,^29–31^ but their application as luminescent materials in optoelectronic devices and their Judd–Ofelt analysis is less explained. Therefore, the authors focused on the optical context and Judd–Ofelt analysis of synthesized Sm^3+^ complexes. Furthermore, the emission and absorption properties, thermal stability, surface morphology, crystalline behaviour, Judd–Ofelt parameter, band gap analysis, relative quantum yield (*η*), intrinsic quantum yield (*ϕ*) and decay time (*τ*) of the synthesized Sm^3+^ complexes were explored in detail. The optical energy gap and geometry optimisation were scrutinized using the ORCA and Avogadro software.

## 2 Experimental part

### 2.1 Materials and methods

Samarium nitrate hexahydrate (Sm(NO_3_)_3_·6H_2_O), the main ligand and secondary ligands used in the synthesis of Sm^3+^ complexes were bought from the commercial dealer Sigma-Aldrich. Barium sulphate and dimethyl sulfoxide (DMSO) were also collected from the above-mentioned vendor. Three fungal strains and four bacterial strains, essentially used in the analysis of antimicrobial properties, were procured from MTCC Chandigarh (Haryana), India.

Various approaches employed to describe the characteristics of as-prepared complexes are described here. A PerkinElmer 400 spectrometer with KBr pellets was used to record the IR spectrum of free (L) and complexes within the 4000 to 400 cm^−1^ range. A Shimadzu UV-3600 plus spectrophotometer was used to record the diffuse reflectance spectra (in powder form) and UV-visible absorption spectra (in liquid phase) of a (L) and all complexes in the range of 200–800 nm with DMSO as the solvent. Using the same instrument, the near-infrared (NIR) region absorption spectra of complexes were obtained over the range of 600–1600 nm. A highly magnified Bruker Avance II 500 NMR spectrometer at 500 MHz frequency was employed to record the ^1^H-NMR and ^13^C-NMR spectra of title complexes and free L in a solution (DMSO + sample), using tetramethylsilane (TMS) as the reference. Carbon (C), hydrogen (H), and nitrogen (N) compositions in all complexes were determined using a PerkinElmer CHN 2400 elemental analyzer. Complexometric titration with EDTA using a xylenol orange indicator was performed to estimate the composition of the Sm^3+^ metal in complexes. The photophysical investigation of complexes in the powder form was performed using a Hitachi F-7000 fluorescence spectrophotometer equipped with a xenon lamp, while the emission and excitation spectra of complexes in the solution phase were recorded using a Horiba Jubin YVON Fluorolog spectrofluorometer (modal FL-3-11). The colorimetric test was carried out precisely using the MATLAB software with the emission data. An analytical instrument, the PerkinElmer STA 600 (with Saturn sensor), was used to analyse the TGA/DTG thermograms of all complexes heated at a rate of 15 °C min^−1^ in a nitrogen atmosphere. The antimicrobial and antioxidant assays were performed by a tube dilution method and a DPPH method respectively. The powder X-ray diffraction (PXRD) patterns for all complexes were scanned using a Rigaku Ultima IV X-ray diffractometer.

### 2.2 Synthesis of C1–C6 complexes

The complexes were synthesized by an eco-friendly and highly efficient liquid-assisted grinding method. L (0.2786 g) and Sm(NO_3_)_3_·6H_2_O metal (0.176 g) taken in a proportion of 3 : 1 were crushed using a pestle and mortar and distilled water was used to make a fine paste for the synthesis of binary complex C1. Few drops of dilute sodium hydroxide solution were added to regulate the pH of the paste. The paste was endorsed to dry in a microwave oven at 45 °C. A dried paste was scratched from the mortar and ground till a white color powder complex was obtained. This powder complex was purified by centrifugation and stored in a sample bottle. Similarly, ternary complexes (C2–C6) were synthesized by following the same procedure as used for the synthesis of the binary complex. In ternary complexes, Sm(NO_3_)_3_·6H_2_O, L and secondary ligands were taken in a proportion of 1 : 3 : 1. To calculate the energy values of the triplet state for L and secondary ligands, gadolinium(iii) complexes were synthesised in a similar manner.^32^

**Scheme 1:**
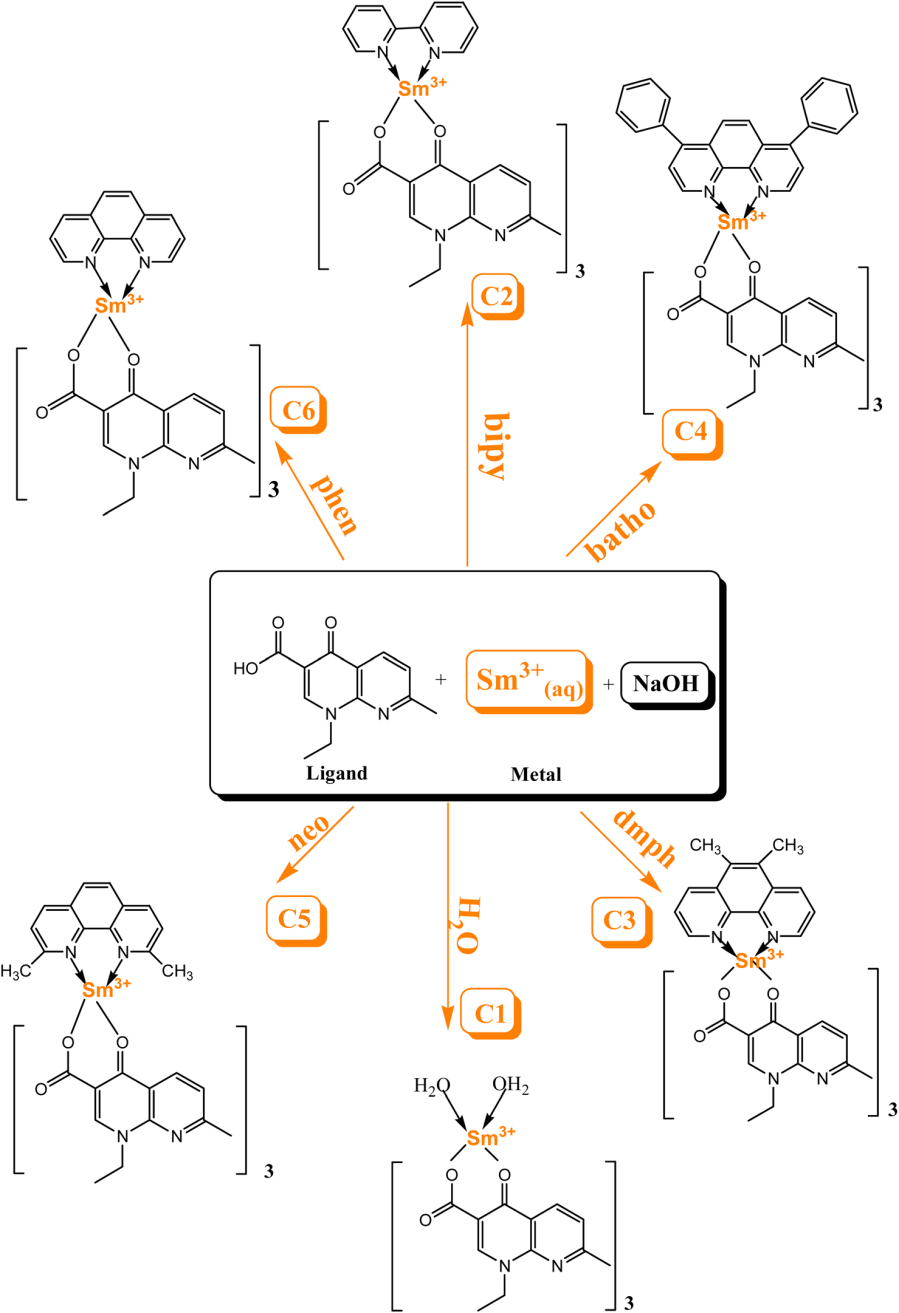
Schematic of the preparation route of Sm^3+^ complexes C1–C6.

Scheme 1 explicates the preparation route of Sm^3+^ complexes C1–C6.^24,33^ The flowchart of the steps of synthesis of C1–C6 complexes by a liquid-assisted grinding method is presented in Fig. S1.†

### 2.3 Biological analysis

#### 2.3.1 Antioxidant activities

The antioxidant properties of the as-prepared complexes were examined by a DPPH (2,2-diphenyl-1-picrylhydrazyl) method, which depends on the scavenging effect of DPPH. The DPPH (free radical) molecule has complete delocalization of odd electrons throughout its structure. For performing the antioxidant experiment, the stock solutions of test samples and standard (ascorbic acid) with different concentrations (100, 75, 50, 25 μg mL^−1^) were prepared in DMSO, and 3 μg mL^−1^ DPPH solution was also prepared in DMSO. Test tubes with 1 mL solution of the corresponding test sample were taken and 1 mL DPPH solution was added into each test tube; the resulting reaction mixtures were kept undisturbed for 30 minutes at room temperature in the darkness for incubation. In the reaction mixture, DPPH reacted with test samples and its purple color was converted into a pale yellow color and a decrease in absorbance was recorded at 517 nm using a UV-visible spectrophotometer. The percentage scavenging activity of DDPH (% inhibition) was evaluated for each mixture according to eqn (S1) given in the ESI†.^34^ Whole experiments were performed in triplicate to get concordant outcomes.

#### 2.3.2 Antimicrobial activities

The broth tube dilution method is a standard and very common approach to interpret the antimicrobial activities of all complexes. Since L is a widely used antibacterial agent, it is used as the standard drug for antibacterial analysis, whereas Griseofulvin is used as the standard drug for antifungal analysis. Four bacterial and three fungal strains were used to test the antimicrobial properties of the complexes. Four pathogenic bacterial strains, two Gram-negative bacteria, namely, *Pseudomonas aeruginosa* (MTCC1688) and *Escherichia coli* (MTCC 443), and two Gram-positive bacteria, namely, *Streptococcus pyogenes* (MTCC442) and *Staphylococcus aureus* (MTCC 96), were used. Three fungal strains *Candida albicans* (MTCC 227), *Aspergillus clavatus* (MTCC 1323) and *Aspergillus niger* (MTCC 282) were included to study the antifungal activity of binary and ternary complexes. The solution of all test samples, reference and L of different concentrations were prepared in DMSO separately in the corresponding test tubes from the stock solution at 2000 μg mL^−1^ concentration. Each test tube was inoculated with bacterial and fungal strains by means of inoculum with size 10^8^ cfu mL^−1^ (cfu mL^−1^ – colony forming unit per millilitre). The strains were mixed by vortex and then incubated in an incubator without agitation.^35^ The minimum concentration of antimicrobial agents that inhibit the microorganism growth was determined in terms of MIC values expressed in micrograms per millilitre.

## 3 Result and discussion

The L and synthesised complexes were characterised by FTIR, ^1^H-NMR, ^13^C-NMR, elemental analysis and UV-vis spectroscopy. Optical characterizations such as emission, excitation spectra, luminescence life time, colorimetry aspect, Judd–Ofelt parameter, quantum efficiency, band gap energy and refractive index were performed successfully. Thermal stability was assessed by the TGA/DTG thermogram and biological behaviour was assessed through antioxidant and antimicrobial activities.

### 3.1 Elemental analysis

The percentage of C, H, N and Sm^3+^ ions, existing in the synthesized complexes C1–C6, was determined using a CHN analyser. The Sm^3+^ ion estimation was accomplished by complexometric titrations using ethylenediamine tetraacetate (EDTA) and xylenol orange as an indicator. The data listed in Table 1 matched well with the calculated values using the proposed molecular formulae mentioned in the introduction section. The FTIR spectra and thermal analysis further confirmed the existence of moisture outside the coordination sphere.

**Table 1:** Elemental analytical data for C1–C6 complexes

Complexes	C (%) found (cal.)	H (%) found (cal.)	N (%) found (cal.)	Sm (%) found (cal.)
C1	46.24 (46.29)	4.56 (4.64)	8.99 (9.00)	16.46 (16.10)
C2	50.85 (50.87)	4.75 (4.71)	10.21 (10.28)	13.80 (13.79)
C3	52.57 (52.62)	4.64 (4.77)	9.90 (9.82)	13.64 (13.79)
C4	56.43 (56.90)	4.50 (4.70)	8.68 (8.85)	11.89 (11.87)
C5	52.57 (52.62)	4.64 (4.77)	9.87 (9.82)	13.64 (13.79)
C6	51.76 (51.78)	4.55 (4.53)	10.00 (10.06)	13.80 (13.49)

### 3.2 Electronic absorption spectra

**Fig. 1:**
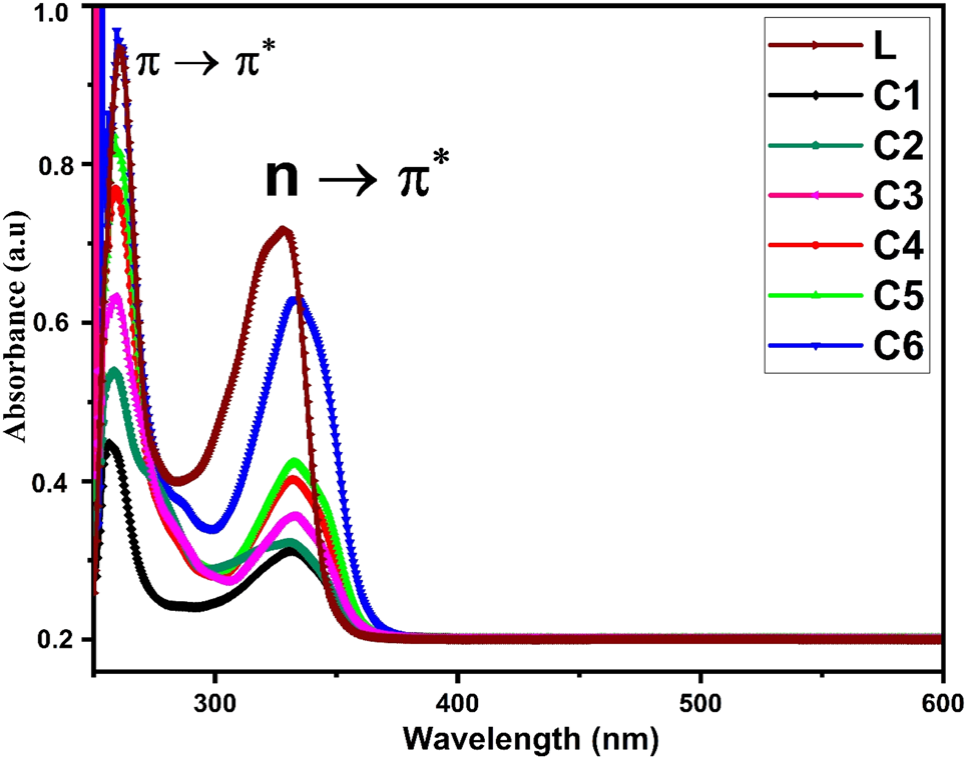
UV-visible absorption spectra of L and C1–C6 in DMSO as the solvent.

Fig. 1 displays the electronic absorption spectra of title complexes and free ligand in DMSO (concentration 10^−5^ mol L^−1^) over the 200–700 nm range. Uncoordinated L has two separate absorption peaks at 240 and 330 nm accredited to π → π* and n → π* transitions, respectively.^36^ The electronic spectra of all complexes also show two absorption bands but with hypochromic and bathochromic shifts. The spectra of complexes indicate the involvement of carbonyl and carboxylic groups in the bonding between L and Sm^3+^ ions.^37^

### 3.3 Spectroscopic assessment

**Fig. 2:**
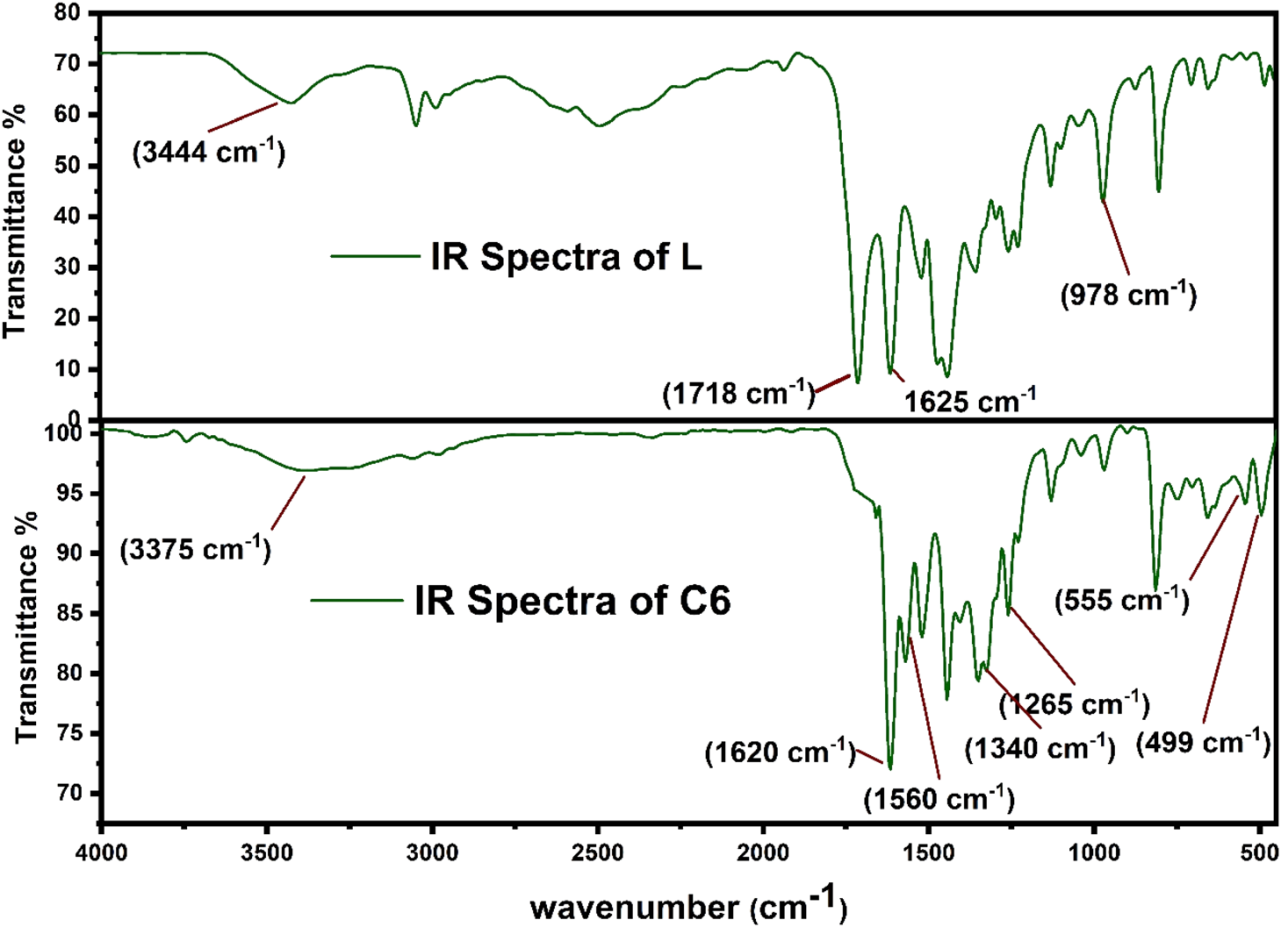
FT-IR spectrum of L and C6 complexes in solid state.

Fig. 2 displays the comparison between the FT-IR spectrum of uncoordinated L and synthesized complex C6. Some considerable peaks are present in both spectra, which further give crucial information about the coordination behaviour of L towards Sm^3+^ ions and bridging behaviour (monodentate and bidentate) of carbonyl (COO^−^) groups. The notable frequencies of IR spectra for all complexes and L are listed in Table 2. The spectra of all complexes resembled one another because of the correlative coordinating approach of Sm^3+^ ions and ligand (L) as portrayed in ESI Fig. S2.† The spectrum of L has a broad band at 3400–3450 cm^−1^ attributed to the *ν*(OH)_carboxylic group_ vibration, and two strong bands have appeared at 1718 cm^−1^ and 1620 cm^−1^, assigned to *ν*(C=O)_carboxylic group_ and *ν*(C=O)_carbonyl group_ present on the pyridine ring. Moreover, the spectra of all complexes did not show any absorption band at 1718 cm^−1^*ν*(C=O), which signified the removal of proton from the COOH group and participation of the carboxyl group in the formation of Sm–O bonds.^38,39^ Slight shifting of the keto pyridine stretching vibration was noticed from 1618 cm^−1^ to 1625–1630 cm^−1^ upon bonding. Decone and Phillips' study gave a criterion to distinguish among the three coordinating sites of the carboxylate group, which suggests that Δ*ν* > 200 cm^−1^ (where Δ*ν* = [*ν*_as_(COO^−^) − *ν*_s_(COO^−^)]) values for monodentate, Δ*ν* < 100 cm^−1^ values are for bidentate or chelating and, finally, Δ*ν* ∼150 cm^−1^ values for the bridging mode of the carboxylate group respectively.^40^ The spectra of synthesized complexes C6 show two characteristic bands, one at 1582 cm^−1^ and the other at 1364 cm^−1^ allotted as *ν*(COO^−^)_asymmetric_ and *ν*(COO^−^)_symmetric_ stretching vibrations of the coordinated carboxylate anion respectively. The observed value of Δ*ν* of the synthesized C1–C6 complexes falls in the range of 200–220 cm^−1^, demonstrating the monodentate binding site of the COO^−^group. Furthermore, two bands in the spectra of complexes at 530–560 cm^−1^ and 485–495 cm^−1^ are ascribed to the vibration of *ν*(Sm–N) and *ν*(Sm–O) bonds respectively, confirming the coordination of L and secondary ligands.^41^ According to the IR analysis, L is co-ordinated to Sm^3+^ ions in bidentate mode *via* one carboxylate and carbonyl oxygen atom, present in the primary ligand.^42,43^

**Table 2:** IR characteristic band for L and C1–C6 complexes^a^

Complexes	*ν*_OH_ COOH, H_2_O	*ν*_OH_ H_2_O	*ν*_COO_−__ asym	*ν*_COO_−__ sym	*ν*_C–O_	*ν*_C–N_	*ν*_Sm–N_	*ν*_Sm–O_
L	3438(b)	—	—	—	1618(s)	—	—	—
C1	—	3445 (b)	1581(m)	1368(s)	1628(s)	—	—	489 (w)
C2	—	3450 (b)	1580(m)	1375(s)	1629(s)	1524 (w)	545 (w)	492 (w)
C3	—	3449 (b)	1577(m)	1371(s)	1627(s)	1525 (w)	531 (w)	489 (w)
C4	—	3451 (b)	1579(m)	1368(s)	1626(s)	1526 (w)	540 (w)	493 (w)
C5	—	3442 (b)	1580(m)	1370(s)	1625(s)	1525 (w)	555 (w)	495 (w)
C6	—	3452 (b)	1582(m)	1364(s)	1628(s)	1528 (w)	560 (w)	494 (w)

a (b) broad, (m) medium (s) sharp (w) weak.

Raman spectra of all the complexes are given in Fig. S3 in the ESI,† which support the coordination site of L in the synthesised complexes. Two strong bands at 1712 cm^−1^ and 1690 cm^−1^ are assigned to the C=O carboxylic and C=O carbonyl group stretching of ligands respectively, as reported in the literature.^44,45^ In the Raman spectra of C1–C6 complexes, the stretching band of the carboxylic group (1712 cm^−1^) is completely vanished and the carbonyl stretching vibration is shifted in a lower wavenumber, which further supports the IR spectra. Two weak bands appeared in the range of 460–513 cm^−1^ and 680–740 cm^−1^ in the Raman spectra of complexes due to (Sm–O)_carbonylic_ and (Sm–O)_carboxylic_ stretching, which indicates that the ligand is binding *via* one carboxylate and carbonyl oxygen atom, present in the primary ligand.^46^

**Fig. 3:**
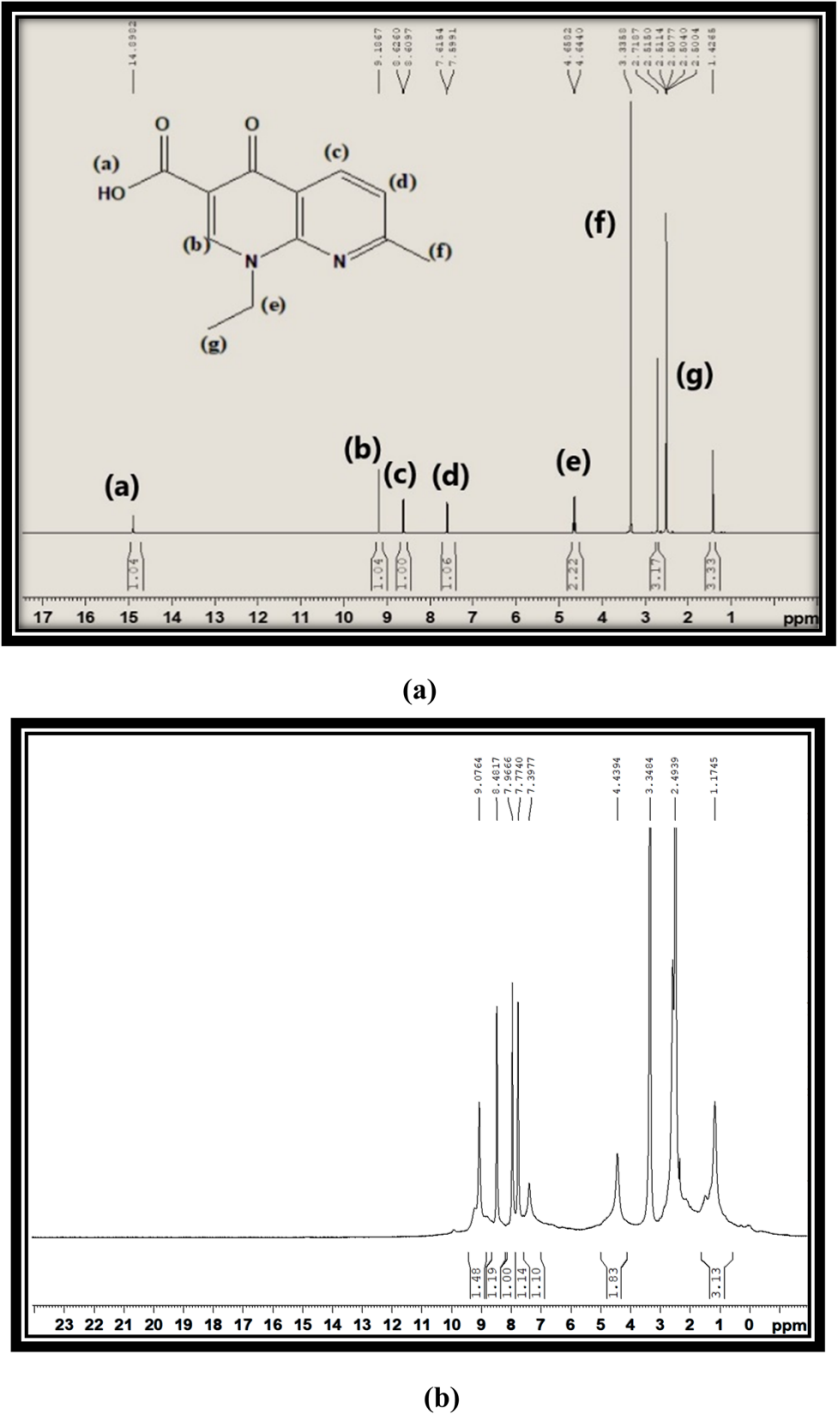
(a) ^1^H-NMR spectra of L and (b) ^1^H-NMR spectra of C6 in deuterated DMSO.

Fig. 3(a) and (b) show the proton NMR spectra of free L and synthesized C6 complex respectively. In the spectrum of free L, a peak at 15 ppm corresponding to the –COOH proton appeared, which completely disappeared in the spectra of synthesized complexes and further confirmed the involvement of the –COOH group in the coordination of L with Sm^3+^ ions. All complexes illustrated the comparable ^1^H NMR spectra, so the spectrum of complex C6 is opted as exemplary of all complexes. Various peaks are present in the spectrum of L at 2.50 ppm, 1.42 ppm and 3.33 ppm due to methyl pyridine, methyl oxo-naphthyridine and ethyl oxo-naphthyridine protons respectively. The aromatic proton signal of L is present in their expected regions of spectrum at 7.60 ppm, 8.61 ppm and 9.18 ppm.^47^ Moreover, a little deviation and broadness in the proton signal shown by the proton NMR spectra of complexes as compared to L is due to the paramagnetic behaviour of Sm^3+^ ions.^48^ ·The comparison between the ^13^C-NMR (DMSO-d_6_) spectra of free L and title complexes is illustrated in Fig. S4(a) and (b).† In the ligand spectrum, the peaks are positioned at 24.93 ppm (CH_3_), 14.90 ppm (CH_3_CH_2_), 46.70 ppm (CH_3_CH_2_), 177.00 ppm (C=O), 108.54 ppm, 118.25 ppm, 122.52 ppm, 135.54 ppm and 149.53 ppm (aromatic rings), and 165.47 ppm (carboxylic acid), while in the spectra of complexes, the peak are positioned at 24.70 ppm (CH_3_), 14.85 ppm (CH_3_CH_2_), 39.58 ppm (CH_3_CH_2_), 150.02 ppm (C=O), 112.74 ppm, 136.46 ppm, 118.63 ppm, and 145.00 ppm (aromatic rings). From the above-mentioned spectral analysis, the carboxylic peak cannot be distinguished, which further confirmed the coordination site of L*via* the COOH group. Additionally, the ketonic peak is significantly shifted to a high field value from 177 ppm to 150.02 ppm, on account of the paramagnetic behaviour of Sm^3+^ ions.^5,49^ The IR, Raman and NMR data of all complexes are correlated and supported with each other.

### 3.4 PXRD and EDAX analysis

**Fig. 4:**
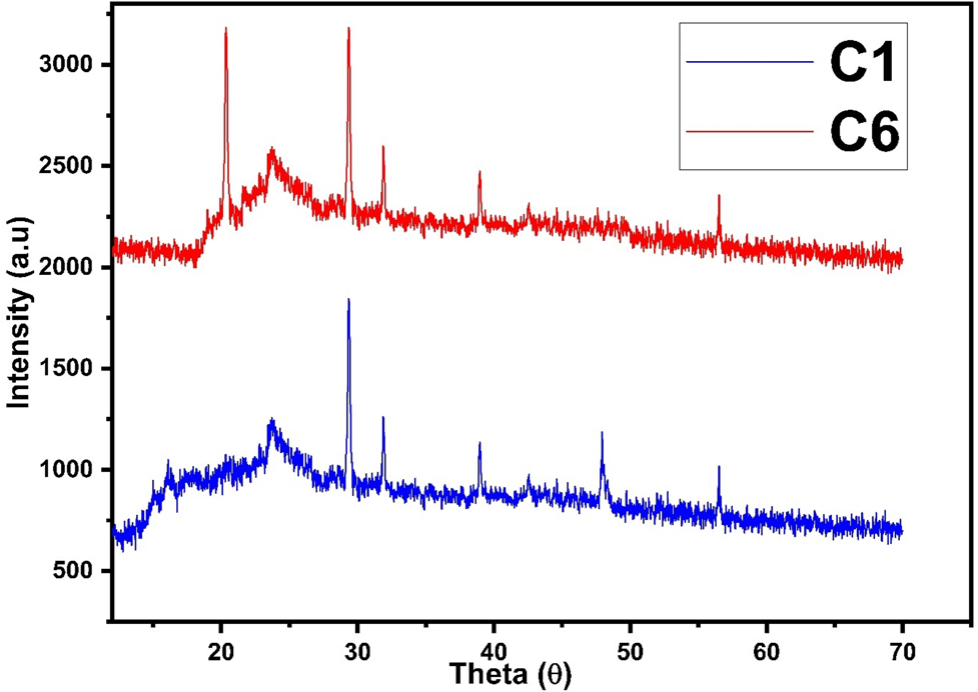
PXRD pattern of C1 and C6 complexes.

The diffraction patterns of complexes (C1 and C6) were recorded at 2*θ* Bragg's angle over the range of 10°–70° using an XRD spectrophotometer at 0.154 nm wavelength. The PXRD patterns of binary and ternary complexes are comparable, thus the C1 and C6 PXRD spectra are given in Fig. 4 and the PXRD spectra of remaining complexes C2–C5 are incorporated in Fig. S5 in the ESI.† Upon deep analysis of diffractogram, the complexes are found to have crystalline behaviour due to sharp peaks observed in the XRD pattern. The particle size (*D*) and delocalisation density (*δ*) of crystalline complexes were estimated by the Debye–Scherrer formulae given below.^50^1
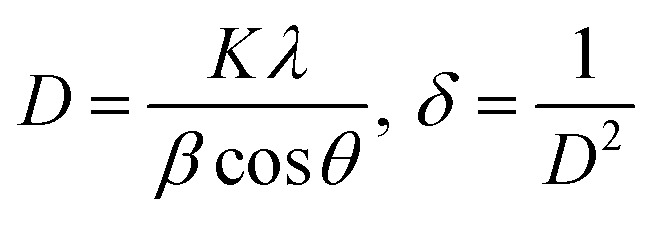


In the aforementioned formulae *λ*, *β* and *θ* are the symbols of wavelength of X-ray (0.154 nm), full width at half maxima of the most dominant peak and peak position respectively. The particle size (*D*) and *δ* values of C1–C6 complexes are 33.8299 nm and 8.7377 × 10^14^ m^−2^, 32.29 nm and 9.59 × 10^14^ m^−2^, 32.04 nm and 9.74× 10^14^ m^−2^, 32.01 nm and 9.76 × 10^14^ m^−2^, 31.33 nm and 10.18× 10^14^ m^−2^ and 30.35 nm and 10.85 × 10^14^ m^−2^ respectively. The particle size of complexes is in the nanometre range; hence, the synthesized complexes can be used as nanomaterials. The higher values of *δ* obtained in our complexes indicate the formation of homogeneous and high-quality complexes.

**Fig. 5:**
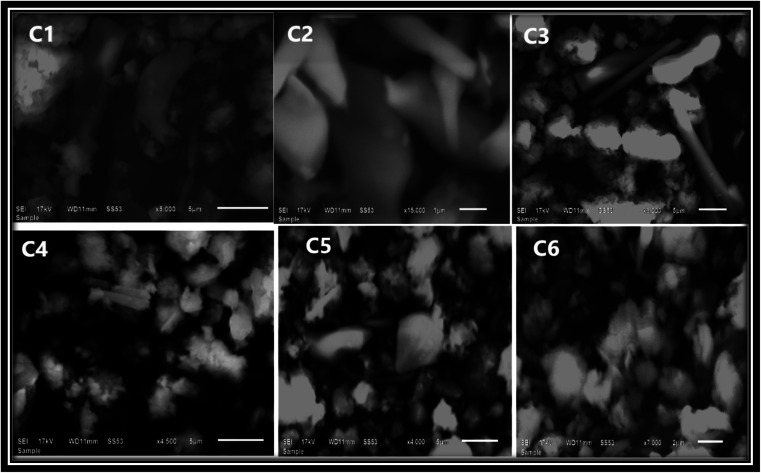
SEM (scanning electron microscopy) image of C1–C6 complexes.

Further, the elemental purity determination was confirmed by EDAX mapping. Fig. S6(a)† shows the EDAX spectrum of all complexes without any additional peaks, thus showing the high purity of the synthesized complexes. Fig. S6(b)† shows the mapping of all complexes, the EDAX mapping reflects that the elements (Sm, C, N, and O) are equally distributed. The compositional analyses claim that the present complexes were synthesized successfully.^51^ The SEM image of all complexes (Fig. 5) reveals the crystalline nature of complexes.^43^

### 3.5 Thermal analysis

**Fig. 6:**
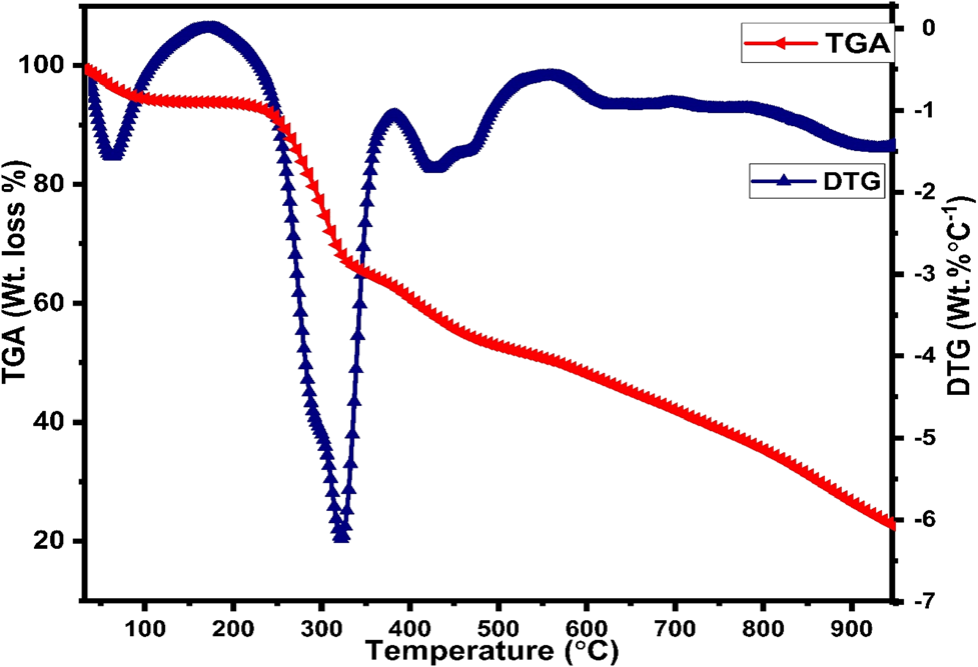
TGA/DTG curve of the C1 complex in a nitrogen atmosphere.

Thermogravimetric (TGA) and differential thermogravimetric (DTG) analysis are vital techniques to investigate the thermal stability of synthesized complexes under an inert atmosphere of nitrogen gas, which is to evade oxidative reactions, whereas the decomposition pattern of all title complexes is comparable to each other, so the thermogram of C6 complex is explained here (Fig. 6) in detail. The thermogram of C1–C5 complexes is given in Fig. S7(a) and (b) in the ESI.† The TG curve of the C6 complex shows three decomposition steps. The first step explicates an initial mass loss of 4.9% (cal. 5.01%) up to 95 °C accredited to the removal of three water molecules existing as water of hydration, which is supported by a DTG minor peak. Further, the complex possesses thermal stability up to 250 °C and after that, the TG curve illustrates a sudden mass loss of 22.17% (cal. 21.90%) from 250 °C to 340 °C temperature range due to the decomposition of one main ligand out of three, which is justified by the strong exothermic peaks in the DTG thermogram at 304 °C. Third inflexion in TG curve depicted a mass loss of 16.18% (cal. 16.21%) up to 480 °C attributed to the decomposition of the secondary ligand from the complex, which is proclaimed by the peak present in the DTG thermogram over the range of 360 °C to 516 °C. The outcomes proclaimed the prominent stability of Sm^3+^ complexes, which is required for their application in display devices. However, the mass of approximately 22%, retained up to 950 °C, could be ascribed to carbon and samarium oxide residues because of the decomposition of the ligand and secondary ligands.^52,53^

### 3.6 Judd–Ofelt studies

**Fig. 7:**
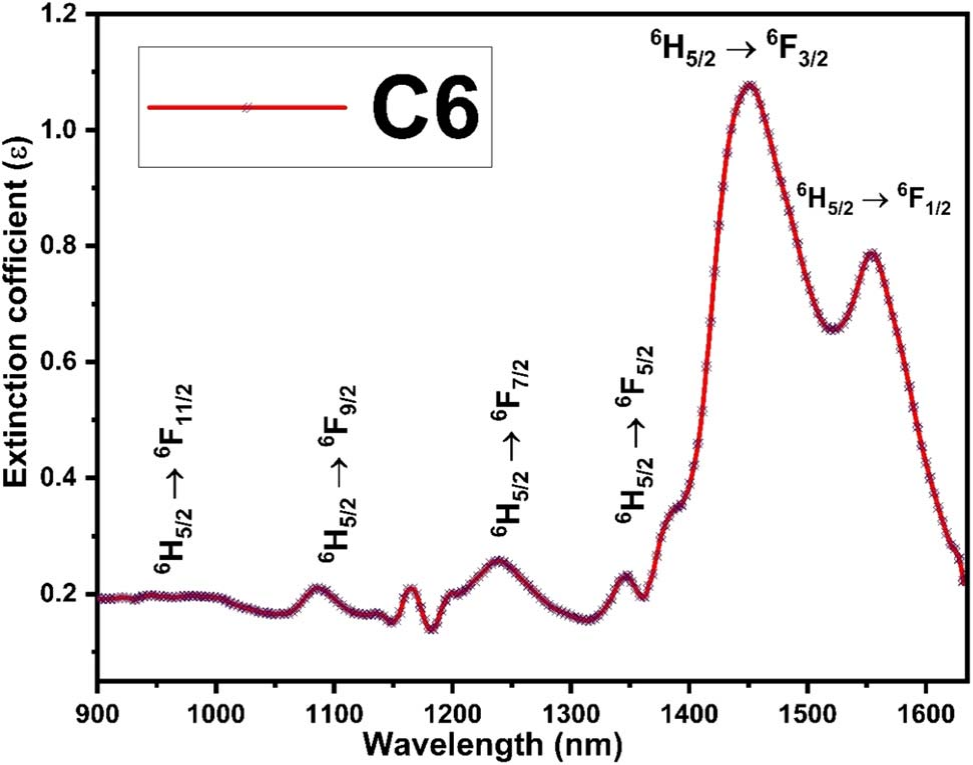
NIR absorption spectra of the C6 complex with different transitions.

The Judd–Ofelt concept is an overwhelming approach to know about the environment around the luminescent centre, symmetry of complexes, degree of covalency and long-range effect.^20^ The Judd–Ofelt intensity parameters (*Ω*_2_, *Ω*_4,_ and *Ω*_6_) of C1–C6 complexes were calculated from the near IR absorption spectra, which were recorded in the solution phase taking DMSO as the solvent. These parameters are utilised to interpret the effect of L on the illuminating properties of Sm^3+^ ions. Some characteristic bands were obtained due to ^6^H_5/2_ → ^6^F_*j*/2_ (*j* = 11, 9, 7, 5, and 1) transitions at 951, 1083, 1240, 1379 and 1454 nm wavelength in the NIR spectra of all complexes. Correspondent nature is shown by the spectra of all complexes, and hence, the spectrum of C6 complex is picked up as exemplary for all complexes and is given in Fig. 7, and the remaining spectra are displayed in Fig. S8 in the ESI.† The crystal field of L present in the surrounding Sm^3+^ ion is responsible for the broadness of peaks and these peaks were allocated according to the ref. 54.

The oscillator strength in the near IR region depends on the extinction (*ε*) coefficient. The experimental oscillator strength (*f*^exp^) for corresponding transition in the NIR region was obtained using the following equation:^55^2
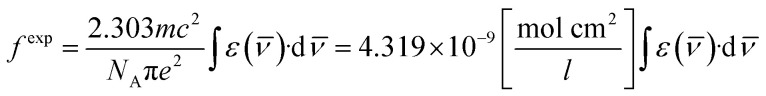
Here, *ε*(ν̄) represents the molar extinction coefficient as a function of wavenumber (cm^−1^) and *c*, *m*, *N*_A_ and *e* stand for the velocity of light, mass of electron, Avogadro's number and charge of electron respectively. Table 3 lists the values of *f*^exp^, which were estimated by taking the area under the specific peak in the absorption spectra.

**Table 3:** Experimental oscillator strength (*f*^exp^ × 10^−6^), calculated oscillator strength (*f*^cal^ × 10^−6^) and root mean square deviation of C1–C6 obtained from different transitions of NIR absorption spectra

Transitions	C1		C2		C3		C4		C5		C6	
^6^H_5/2_ →	*f* ^exp^	*f* ^cal^	*f* ^exp^	*f* ^cal^	*f* ^exp^	*f* ^cal^	*f* ^exp^	*f* ^cal^	*f* ^exp^	*f* ^cal^	*f* ^exp^	*f* ^cal^
^6^F_1/2_	4.61	4.65	5.33	5.30	5.58	5.62	6.70	6.79	6.66	6.67	7.02	7.11
^6^F_5/2_	1.75	1.54	2.09	1.98	2.98	2.73	3.80	3.50	2.63	2.01	4.12	3.58
^6^F_7/2_	3.02	3.50	4.44	4.67	7.82	8.30	10.21	10.86	5.53	6.90	5.93	7.10
^6^F_9/2_	3.25	2.85	3.88	3.78	7.40	6.80	9.61	8.85	6.38	5.90	6.09	5.50
^6^F_11/2_	1.22	0.50	1.26	0.64	1.53	1.16	1.29	1.50	1.18	1.00	1.07	0.90
*δ*_rms_ × 10^−6^	0.146		0.1042		0.0705		0.0665		0.14232		0.121	

Estimated oscillator strength (*f*^cal^) for electric dipole transition explored from the fundamental excited state is described in the following equation:3
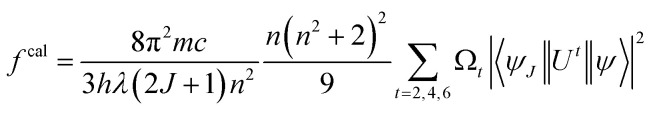


In eqn (3), *λ*, *J* and *n* signify the wavelength of the peak for the corresponding transition, total angular momentum of ground state and refractive index of complexes respectively, whereas *Ω*_*t*_ (*t* = 2, 4, and 6) is the Judd–Ofelt intensity parameter and 
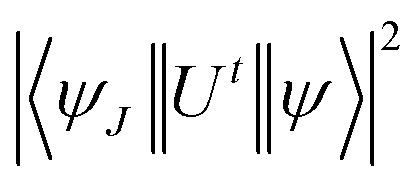
 is the reduced squared matrix element independent of L. In the oscillator strength (*f*^cal^) calculation, magnetic transitions in absorption spectra were neglected because they are very less intense.

Theoretical and calculated oscillator strengths were harmonised (*f*^exp^ = *f*^cal^) to achieve a set of linear equations for each transition such as eqn (S2) given in the ESI.† The obtained set of equations were simplified by the least-square fitting method, and intensity parameters were obtained. Further employing the Judd–Ofelt parameter in eqn (3), the calculated oscillator strength (*f*^cal^) was investigated. The root mean square deviation (*δ*_rms_) is a parameter to justify the accuracy of fitting approach and it was determined by the following relation:^33,56^4
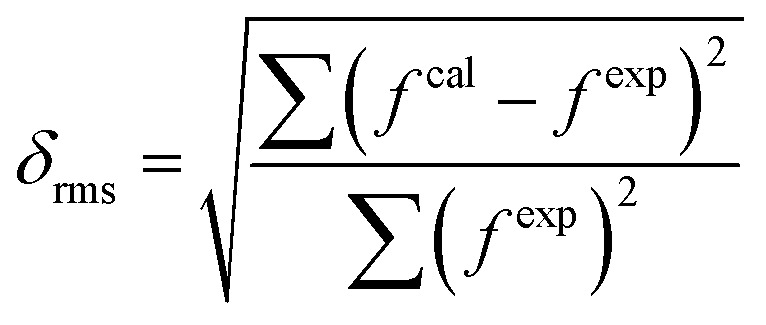
where *f*^exp^ and *f*^cal^ represent the experimental and calculated oscillator strengths respectively. Lesser values of *δ*_rms_ demonstrate excellent fitting of experimental and calculated oscillator strengths. The values of *f*^exp^, *f*^cal^ and *δ*_rms_ are tabulated in Table 3. The ^6^H_5/2_ → ^6^F_3/2_ transition was omitted in the investigation process of Judd–Ofelt parameters due to its abnormal behaviour.

The resulting intensity parameters of complexes C1–C6 are compared with other existing parameters reported in the literature. The JO parameters of the present complexes obey the pattern *Ω*_2_ > *Ω*_6_ > *Ω*_4_.^57^ The *Ω*_2_ parameter is very sensitive to the factor, which affects the surrounding of Sm^3+^ ions such as degree of covalency and symmetry of ligand field and structure of complexes. The *Ω*_4_ and *Ω*_6_ parameters display the rigidity and viscosity of Sm^3+^ complexes, and depend upon their dielectric properties.^58,59^ A larger value of *Ω*_2_ illustrates a lower symmetry and a greater covalence character, whereas higher *Ω*_4_ and *Ω*_6_ display decent rigidity in the C1–C6 complexes. Most intense peak in the emission spectra of complexes shows stark splitting due to the non-cubic symmetry, which is further supported by the *Ω*_2_ intensity parameter. However, the parameters of synthesized complexes show a greater value than that of other reported complexes reported in the literature, as encapsulated in Table 4.

**Table 4:** Judd–Ofelt parameter and their trend observed in different [Sm(ligand)_3_·secondary] complexes reported in the literature and C1–C6 complexes

Sm^3+^ complexes	*Ω*_2_ × 10^−20^	*Ω*_4_ × 10^−20^	*Ω*_6_ × 10^−20^	Trend	Reference
C1	15.53	1.32	3.70	*Ω*_2_ > *Ω*_6_ > *Ω*_4_	This work
C2	17.88	1.96	4.88	*Ω*_2_ > *Ω*_6_ > *Ω*_4_	This work
C3	18.36	3.41	8.82	*Ω*_2_ > *Ω*_6_ > *Ω*_4_	This work
C4	22.53	4.54	11.45	*Ω*_2_ > *Ω*_6_ > *Ω*_4_	This work
C5	22.58	1.44	7.72	*Ω*_2_ > *Ω*_6_ > *Ω*_4_	This work
C6	23.75	4.54	6.92	*Ω*_4_ > *Ω*_6_ > *Ω*_2_	This work
[Sm(tta)_3_(H_2_O)_2_]	2.24	4.4	2.3	*Ω*_4_ > *Ω*_6_ > *Ω*_2_	58
[Sm(tta)_3_(tppo)_2_]	2.9	7.9	3.7	*Ω*_4_ > *Ω*_6_ > *Ω*_2_	58
[Sm(tta)_3_phen]	0.63	3.1	2.0	*Ω*_4_ > *Ω*_6_ > *Ω*_2_	58
[Sm(tta)_3_bipy]	4.2	11.2	8.5	*Ω*_4_ > *Ω*_6_ > *Ω*_2_	58
[Sm(S_2_PPh_2_)_3_(THF)_2_]	25.49	6.88	6.11	*Ω*_2_ > *Ω*_4_ > *Ω*_6_	60
[Sm(Se_2_PPh_2_)_3_(THF)_2_]	13.11	6.50	6.26	*Ω*_2_ > *Ω*_4_ > *Ω*_6_	60
[Sm(L)_3_bipy]	17.6	3.86	3.86	*Ω*_2_ > *Ω*_4_ > *Ω*_6_	20
[Sm(L)_3_phen]	17.0	6.92	2.91	*Ω*_2_ > *Ω*_4_ > *Ω*_6_	20
[Sm(DBM)_3_phen]	0.13	0.035	0.042	*Ω*_2_ > *Ω*_6_ > *Ω*_4_	57
[Sm(DBM)_3_(topo)_2_]	0.18	0.050	0.026	*Ω*_2_ > *Ω*_4_ > *Ω*_6_	57
[Sm(DBM)_3_(tppo)_2_]	0.31	0.040	0.061	*Ω*_2_ > *Ω*_6_ > *Ω*_4_	57
[Sm(sal)_3_phen]	7.9	7.0	1.2	*Ω*_2_ > *Ω*_4_ > *Ω*_6_	61

The total radiative rate (*A*_rad_) of C1–C6 complexes under the irradiation of UV-vis light is the sum of all radiative transition probabilities (electric and magnetic dipole transition) for the ground state to the excited state. The *A*_rad_ values were determined employing the Judd–Ofelt values in the following relation:5
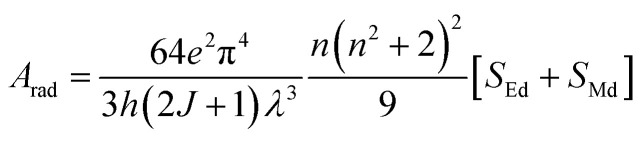
Here, *S*_Md_ denotes the magnetic dipole line strength and *S*_Ed_ stands for the electric dipole line strength. In the computational process, *S*_Md_ is ignored and the equation is expressed as eqn (S3) in the ESI.†*S*_Ed_ is the product of intensity parameter and reduced square matrix element, which is represented in term given below and the matrices were taken from Monteiro *et al.*^58^6
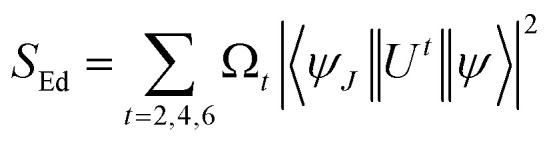


The values (*A*_rad_) for all the synthesized complexes are tabulated in Table 5. An upsurge observed in radiative values of C2–C6 than the C1 complex is the evidence that the introduction of secondary ligands in C2–C6 complexes by substituting water molecules increases the luminescence intensity by diminishing the non-radiative decay.

**Table 5:** Radiative transition rate (*A*_rad_), nonradiative transition rate (*A*_nrad_), experimental decay time (*τ*_exp_), radiative decay time (*τ*_rad_) and intrinsic quantum yield (https://www.sciencedirect.com/topics/chemistry/quantum-yield) (*ϕ*%) in the solid state of C1–C6 complexes

Complexes	*A*_rad_ (s^−1^)	*A*_nrad_ (s^−1^)	*A*_total_	*τ*_exp_ (ms)	*τ*_rad_ (ms)	*ϕ* (%)
C1	338.31	430.92	769.23	1.30	2.955	43.98
C2	410.97	303.31	714.28	1.40	2.433	57.53
C3	528.42	125.17	653.59	1.53	1.892	80.84
C4	520.13	93.36	613.49	1.63	1.922	84.78
C5	660.34	90.01	750.10	1.33	1.515	88.03
C6	600.60	66.00	666.60	1.50	1.666	90.09

### 3.7 Optical band gap and Urbach energy

To study the optical characteristics of prepared complexes, the band gap value was measured by a meticulous reflectance (DR) spectral probe, which was recorded in solid state with barium sulphate as the reference in the 200–800 nm range. Kubelka–Munk's (K–M) hypothesis was put into operation on reflection data and solved according to Tauc's equation^62^ and the optical energy band gap outcomes were gained as follows:

7 [*F*(*R*_∞_)*hν*]^1/*n*^ = *C*(*hν* − *E*_g_)

**Fig. 8:**
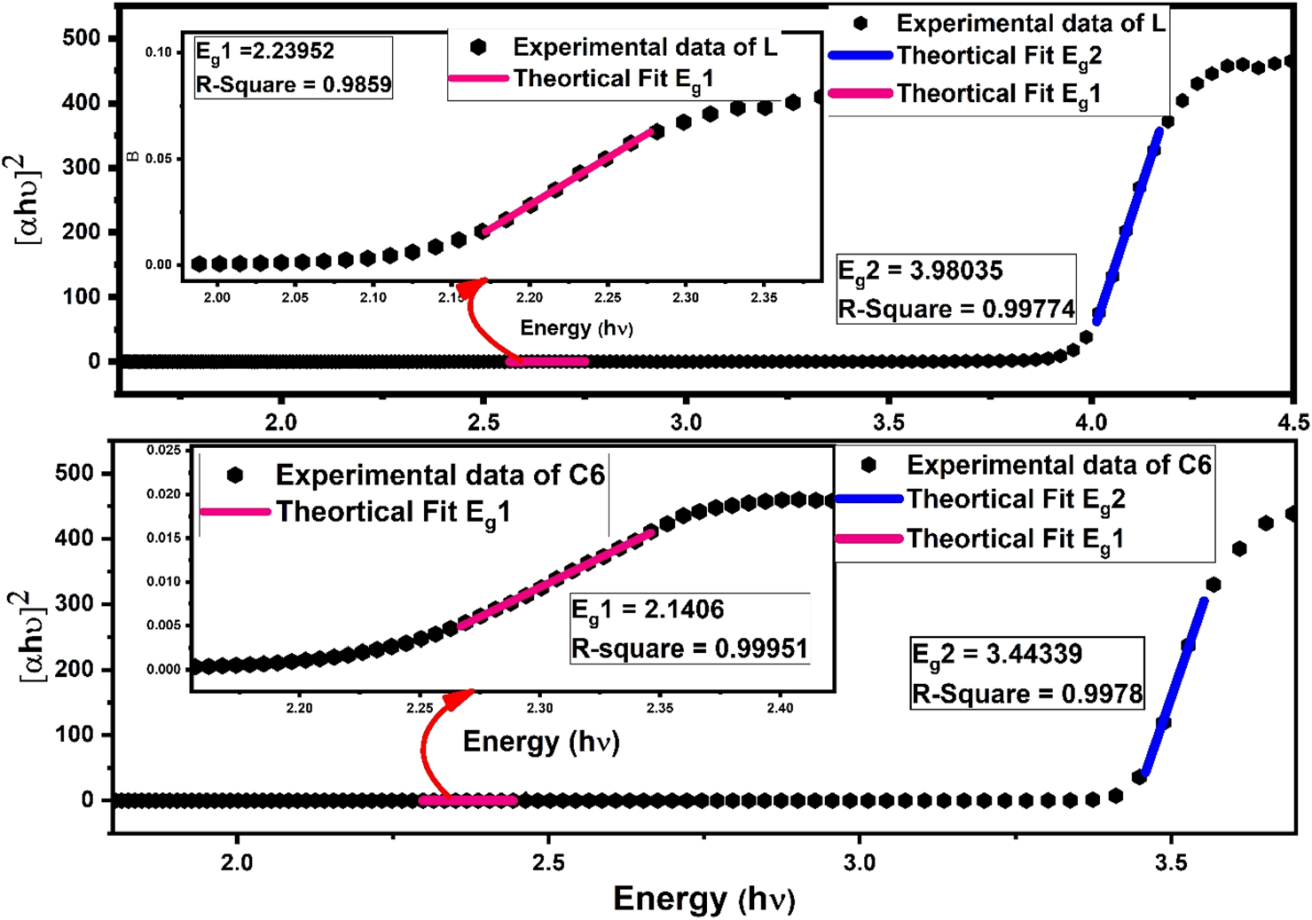
Linear fitted curve of L and C6 using Tauc's relation to the obtained band gap values (*E*_g2_) in a higher energy region; the inset of picture shows the zoomed lower energy region with another band gap (*E*_g1_).

In eqn (7), *C* is a constant, *hν* symbolise photon energy, while *n* is the parameter to give information about the nature of electronic transition (*viz.* if *n* = 1/3, 3 indicates the direct or indirect forbidden transition, and if *n* = 1/2, 2 indicates the direct or indirect allowed transition), which occurs *via* an absorption process. Some other supporting eqn (S4) and (S5) are given in the ESI.† By dint of linear fitting, more than one optical band gap (*E*_g_) values were obtained experimentally for the indirect allowed transition (*n* = 2) in L along with complexes.^5,63^ One band gap is in the lower energy (*E*_g1_) region and another is in the higher energy region (*E*_g2_), as depicted in Fig. 8 for the C6 complex, whereas Fig. S9 in the ESI† displays the linear fitting curve to obtain two band gaps of C1–C5 complexes. The adjacent *R*-square (*R*^2^) values explain the validity of fitting between experimental data and theoretical fitting. The observed *R*^2^ value is >0.99, indicating that the excellent fitting was achieved. The values of optical band gap (*E*_g1_ and *E*_g2_) of the synthesised complexes lower than that of free L demonstrate the decrease in energy gap between HOMO and LUMO energy levels due to the inclusion of additional energy state in the band gap by Sm^3+^ ions in complexes.^63^ A slight decrease in (*E*_g1_ and *E*_g2_) values for the ternary complexes could be due to the secondary ligand. The band gap energy values (*E*_g1_ and *E*_g2_) and adjacent *R*-square (*R*^2^) of L and all complexes are documented in Table 6.^64–66^

**Table 6:** Compiled parameter of the optical analysis of L and complexes C1–C6^a^

	Urbach energy			Band gap analysis				Refractive index (*n*)
	Slope	*U*_e_ (meV)	*R* ^2^	*E*_g1_ (eV)	*R* ^2^	*E*_g2_ (eV)	*R* ^2^	
L	53.18	18.80	0.9992	2.23	0.994	3.98	0.9977	1.883
C1	14.96	66.84	0.9992	2.20	0.999	3.45	0.9974	1.950
C2	13.03	76.71	0.9948	2.14	0.998	3.46	0.9980	1.951
C3	10.32	96.89	0.9945	2.17	0.998	3.44	0.9980	1.952
C4	8.54	117.08	0.9969	2.19	0.999	3.42	0.996	1.953
C5	7.68	130.19	0.9938	2.15	0.999	3.40	0.996	1.954
C6	6.44	155.27	0.9923	2.14	0.999	3.40	0.997	1.954

a *E*_g1_ and *E*_g2_ = optical band gap values for lower and higher energy regions respectively; *U*_e_ = Urbach energy; *R*^2^ = best fit parameter for the respective fitting; *n* = refractive indices.

Additionally, more than one band gap value existing in any system accredited the two different charge transfer processes taking place. The optical band gap values of synthesised complexes are consistent with the range of semiconductor (2–4 eV) devices, thus these complexes possess good contendership for photovoltaic cells, laser application and solar cell. Furthermore, since any system with two or more band gaps will result in an efficient conversion of solar energy, these complexes can have impressive materiality in solar cells.^67–69^

The dependency of the optical band gap energy on the refractive index (*n*) values of L and complexes is described by the following relation:8
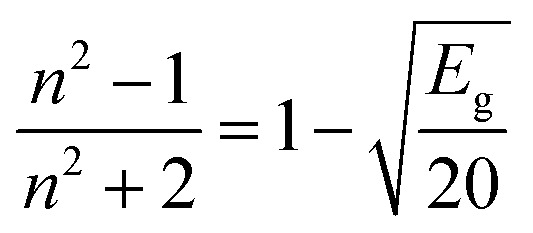


The outcomes of refractive index seem close to semiconducting metal oxides and are tabularised in Table 6; hence, these complexes have applications in optoelectronic devices.

Further, another optical parameter, width of the Urbach tail existing in the lower energy region,^70^ was also calculated by taking the exponential absorption coefficient on the energy under the Urbach rule^71,72^ given mathematically as follows:

9 *α* = *α*_o_ exp(*hν*/*U*_e_)

**Fig. 9:**
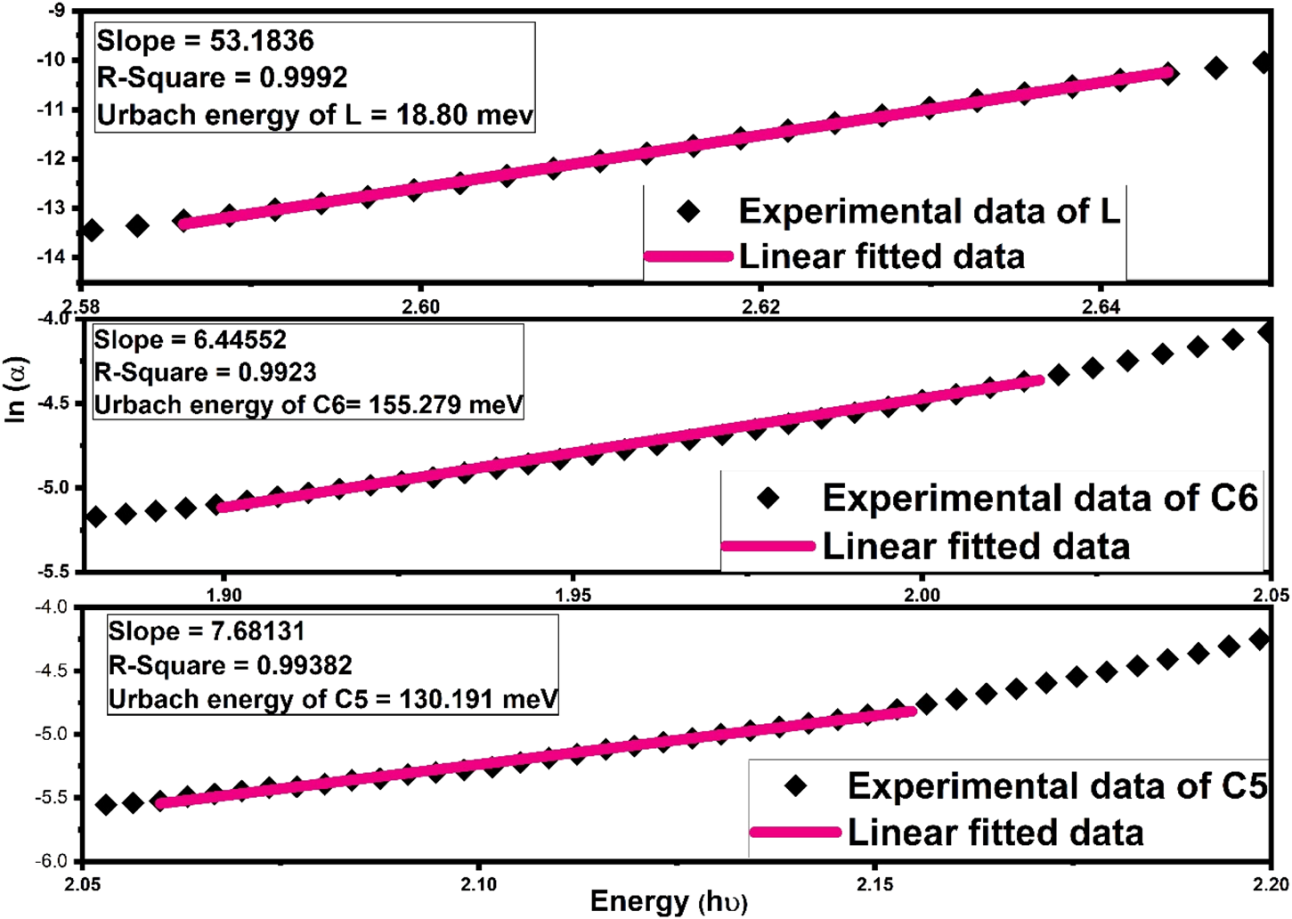
Fitting of the absorption coefficient of L, C6 and C5 samarium complexes using a new empirical rule to the obtained Urbach band tail width (*U*_e_).

Here, *α*_o_ is the optical constant and *U*_e_ denoted the Urbach energy. The Urbach energy manifests the disorders and defect levels in the forbidden band gap zone. The *U*_e_ (Urbach energy) calculation was accomplished by the inverse of slope of linear portion existing in a lower energy domain in the graph between ln(*α*) and photon energy (*hν*) represented in Fig. 9 (L and C6, C5) and Fig. S10† (C1–C4).^73^ The calculated values of Urbach energy for all complexes are listed in Table 6. The increased Urbach tail for all complexes relative to free L indicated that the energetic disorder and structural defect are increased due to the insertion of Sm^3+^ ions.^5^ Urbach energy outcomes follow the inverse trend of band gap values, which were compatible for Al-doped ZnO films.

### 3.8 PL analysis

**Fig. 10:**
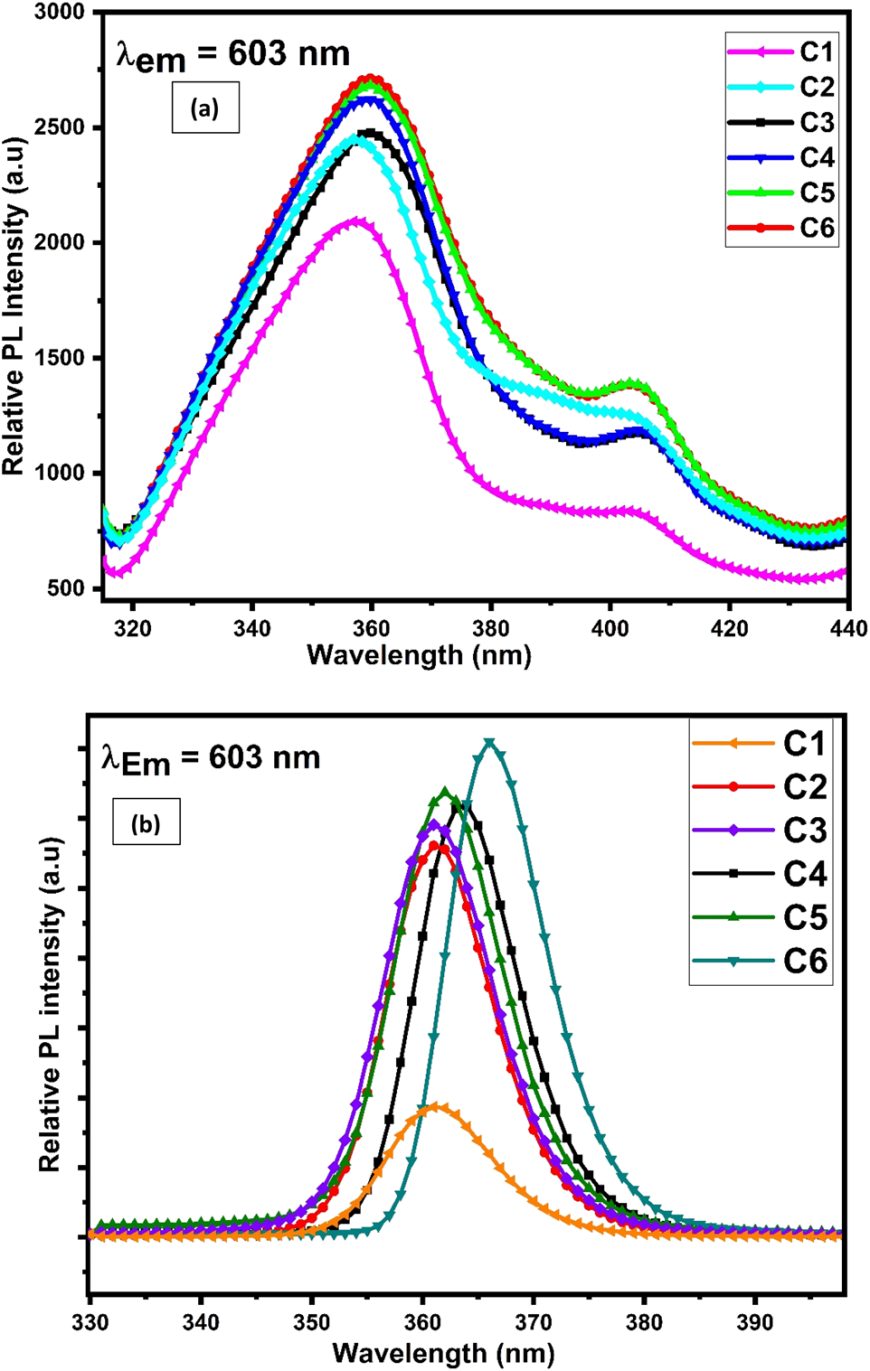
Photoluminescence excitation spectra of C1–C6 complexes in (a) solid phase and (b) 10^−5^ M solution of complexes in DMSO by observing the emission wavelength at 603 nm.

Excitation and emission spectra of synthesised complexes are recorded using a photoluminescence spectrophotometer in the solid form within 200–500 nm and 400–800 nm wavelength range respectively. The excitation spectra were recorded in the solid phase of complexes C1–C6, by monitoring emission at 603 nm. Fig. 10(a) displays the excitation spectra of all complexes in the solid state, which shows two broad bands centered at 360 and 404 nm. These two significant excitation peaks in the solid state account for π–π* and n–π* transition of L respectively.^74^

The solution-phase excitation spectra (Fig. 10(b)) portrayed a band between 350 and 385 nm centered at 362 nm, which is credited due to the π–π* transition of L and is less broad than that in the solid state. It is noteworthy that the excitation band appeared in the near-UV region, which suggested that the complexes are completely excited in the UV domain and are applicable in UV-LEDs.^75^

**Fig. 11:**
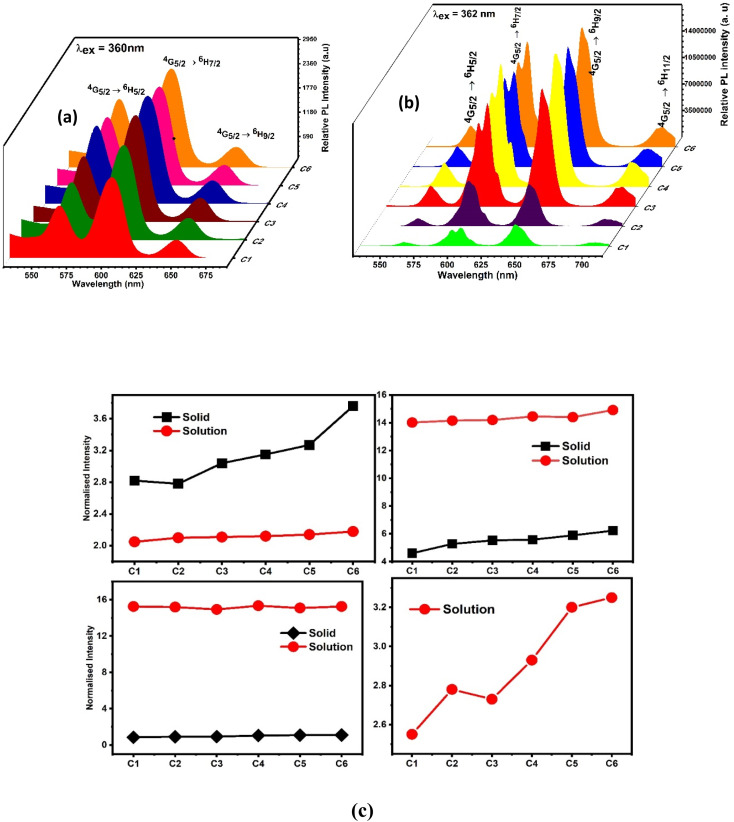
Three-dimensional photoluminescence emission spectra of (a) solid-phase complexes and (b) 10^−5^ solution of complexes in DMDO recorded at 360 nm and 362 nm excitation wavelengths respectively. (c) Comparison between the normalised emission peak intensities of C1–C6 complexes in the solid phase and solution with DMSO as the solvent.

However, the emission spectra describe the illuminating properties of the synthesised complexes, as depicted in Fig. 11(a) (powder form) and Fig. 11(b) (solution form). Upon irradiation with 360 nm and 362 nm UV light, the complexes showed well-defined typical emission spectra in solid and solution phases respectively. The emission spectra in the solid phase comprised three significant peaks of Sm^3+^ ions at 565 nm, 603 nm and 650 nm designated to ^4^G_5/2_ → ^6^H_5/2, 7/2, 9/2_ electronic transition respectively, whereas the solution phase spectra comprised four significant peaks of Sm^3+^ ions at 563 nm, 605 nm, 646 nm and 704 accredited due to the ^4^G_5/2_ → ^6^H_5/2, 7/2, 9/2, 11/2_ electronic transition respectively. The electronic transitions such as ^4^G_5/2_ → ^6^H_5/2,_ Δ*J* = 0, ^4^G_5/2_ → ^6^H_7/2_, Δ*J* = ± 1 and ^4^G_5/2_ → ^6^H_9/2_, _11/2_, Δ*J* = ± 2 followed the total angular momentum selection rule. Comparison between the intensity ratio of all transitions in solid and solution phases of complexes in the emission spectra is disclosed in Fig. 11(c). The bright orange luminescence rendered by the complexes confirmed the hypersensitive ^4^G_5/2_ → ^6^H_7/2_ (magnetic with dominant electric) transition in the solid state, while in the solution phase, complexes rendered reddish-orange color due to ^4^G_5/2_ → ^6^H_9/2_ transition which is more intense than ^4^G_5/2_ → ^6^H_7/2_ transition. The emission profile of complexes justified the applicability of these complexes in orange- and reddish-orange-emitting devices. Stark splitting perceived in the emission spectra of the complexes in the solution phase indicates their non-cubic symmetry in the surrounding of Sm^3+^ ions, which is further proved by the Judd–Ofelt intensity parameters and intensity ratio (*I*_Sm_).^24,76^ Stark splitting would be justified by the Gaussian fitting, which deconvoluted the emission transition into four peaks, as shown in Fig. S11.† It is worthy to note that in the solution-phase emission spectra, within the range of 450–500 nm, no ligand base emission is observed, which indicated the effective sensitization of metal ions by the ligand. The upsurge observed in the sensitization efficiency obtained for ternary (C2–C6) complexes demonstrates the replacement of water molecules (binary complex C1) with the secondary ligand. This secondary ligand has synergistic effects with L, which leads to effective energy transfer.^77^

To explain the surrounding of the Sm^3+^ ion, the intensity ratio (*I*_Sm_) was calculated by dividing the electric dipole intensity by the magnetic dipole intensity. The higher the intensity ratio, the lower the symmetry around the luminescent centre and *vice versa*.^49^ The outcomes of *I*_Sm_ for all complexes are lesser than one, as listed in Table 9; thus, the complexes are applicable for photonic devices in the solid state. However, in the solution phase, the *I*_Sm_ values come out to be higher in magnitude, suggesting asymmetric surrounding of the luminescent centre.^78,79^ The observed values of *I*_Sm_ (Table 10) in the solution phase are more than one and in the range of 6.93–10.1.

### 3.9 Decay time and radiative properties

**Fig. 12:**
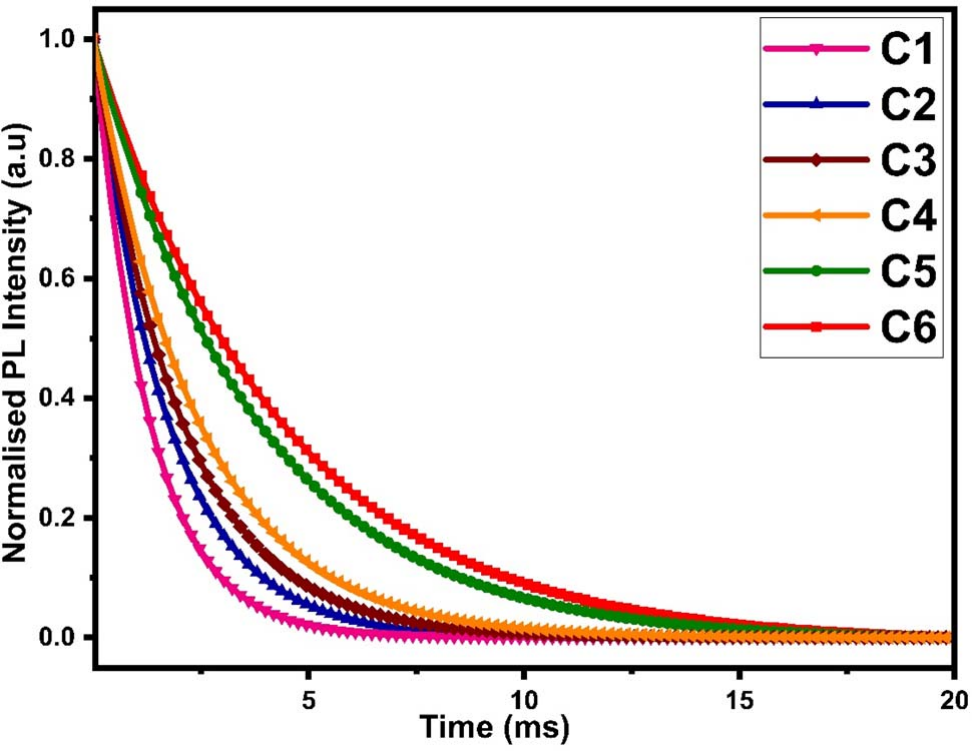
Photoluminescence decay curve of C1–C6 at room temperature.

The luminescence decay time data of all complexes were obtained by fixing the excitation and emission wavelengths at 360 nm and 603 nm respectively. The outcome of decay curve was found by nonlinear curve fitting with a single exponential function, as depicted in Fig. 12 and the decay time values were mathematically evaluated by the following relation:

10 *I* = *I*_0_ exp(−*t*/*τ*_exp_)

Here, *I* represents the emission intensity of peaks at time *t*, *I*_0_ symbolizes the emission intensity of peaks at time *t* = 0 and *τ*_exp_ denotes the experimental decay life time. The experimental decay curve of all complexes portrayed the homogeneous surrounding of Sm^3+^ ions, as depicted in Fig. 12. The decay life time (*τ*_exp_) and radiative (*A*_rad_) and non-radiative (*A*_nrad_) rates are linked and rationalised by eqn (S6) given in the ESI.† The normalized decay curve obeys the mono exponential behaviour, which is significant indication of the presence of the mono luminescent centre (Sm^3+^ ion), and this is in excellent agreement with the phase-probe findings. Besides, it can be observed that computed decay time is higher than the already discussed [Sm(ligand)_3_·secondary] complexes from the literature, as presented in Table 11. The higher decay time of our complexes could be the consequence of extensive conjugation in L. The solvent molecule was substituted by a secondary ligand in ternary complexes, so the value of decay time was enhanced in ternary complexes, as compared to binary complexes.

In addition, the radiative decay time values of the synthesized complexes were derived using the total radiative rate, as specified by the following relation:11
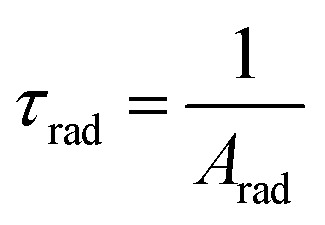
*A*_rad_ is the sum of all radiative decay rates of each transition commencing from the excited state (^4^G_5/2_) to the ground state.

The intrinsic quantum yield *ϕ* (%) is the ratio of total emitted energy (radiative and non-radiative) form to the emitted energy in the radiative form of synthesized complexes, which expresses the optical properties of Sm^3+^ ions. *ϕ* (%) was determined according to the following equation:^20^12
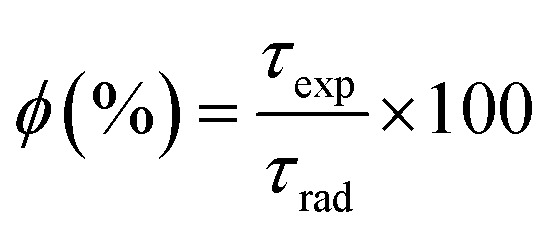
where *ϕ* refers to the quantum yield, and *τ*_exp_ and *τ*_rad_ represent the experimental decay time and natural decay. Further, the fluorescence quantum yield (*η*) of Sm(iii) complexes was also derived using quinine sulphate in dilute sulphuric acid as a reference using the following relation:^80^13
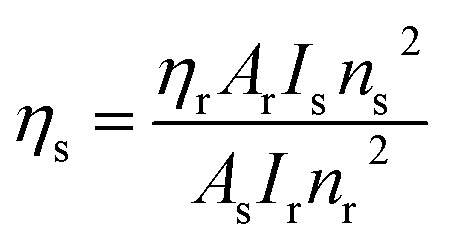
In eqn (13), *η*_s_ and *η*_r_(0.546) stand for the quantum yield of the sample and reference respectively.^80^*I*_s_, *A*_s_, *I*_r_, and *A*_r_ represent the integrated emission intensity, absorbance at the excitation wavelength of the complexes, integrated emission intensity, and absorbance at the excitation wavelength of reference respectively. *n*_s_ and *n*_r_ (1.33) are symbols of the refractive index of samples and reference respectively.

Table 9 presents the data of luminescence quantum yield and Table 10 the values of relative quantum yield of C1–C6 complexes. A significant upsurge was perceived in both the quantum yield values for complexes with secondary ligands C2–C6 relative to complexes with water molecules due to a lower nonradiative energy decay and enhanced luminescence. The intensification in luminescence in ternary complexes is due to the co-operative effect of secondary ligands. It is worth taking a look that the outcomes of these parameters are higher than the previously reported similar [Sm(ligand)_3_·secondary ligand] complexes present in the literature, as given in Table 7.

**Table 7:** Intrinsic and relative quantum yields (https://www.sciencedirect.com/topics/chemistry/quantum-yield) of our complexes compared with other [Sm(ligand)_3_·auxillary] complexes reported in the literature^a^

Complex	*ϕ* (%) (solid state)	*η* (%) (solution)	Reference
[Sm(L)_3_·(H_2_O)_2_]	43.98	35.88	Present work
[Sm(L)_3_·bipy]	57.53	53.47	Present work
[Sm(L)_3_·dmph]	80.84	53.68	Present work
[Sm(L)_3_·batho]	84.78	59.31	Present work
[Sm(L)_3_·neo]	88.03	55.03	Present work
[Sm(L)_3_·phen]	90.09	67.27	Present work
[Sm(L)_3_·(H_2_O)_2_]	23.58	1.93	5
[Sm(L)_3_·dmph]	33.05	2.32	5
[Sm(L)_3_·bipy]	39.44	2.67	5
[Sm(L)_3_·phen]	47.01	15.67	5
[Sm(L)_3_·batho]	50.95	19.86	5
[Sm(L)_3_·Mphen]	18.29	2.09	84
[Sm(L)_3_·Biq]	18.51	6.77	84
[Sm(L)_3_·Bathocup]	21.51	15.38	84
[Sm(L)_3_·2H_2_O]	14.29	1.23	77
[Sm(L)_3_·Phen]	14.58	1.84	77
[Sm(L)_3_·Bipy]	15.96	2.16	77
[Sm(L)_3_·Neo]	17.61	3.27	77
[Sm(L)_3_·Batho]	17.74	6.62	77
[Sm(fod)_3_·tptz]	2.46	—	85
[Sm(fod)_3_·impy]	8.27	—	85
[Sm(fod)_3_·indazole]	1.18	—	85
[Sm(fod)_3_·(tppo)_2_]	1.64	—	85
[Sm(L′)_3_·Phen]	—	1.98	86
[Sm(L′)_3_·Bipy]	—	2.46	86
[Sm(L′)_3_·Neo]	—	6.41	86
[Sm(L′)_3_·Bathophen]	—	13.93	86

a *ϕ* = intrinsic quantum yield obtained as 
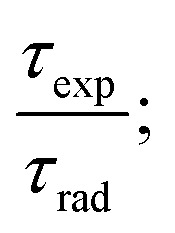
*η* = relative quantum yield taking quinine sulphate (https://www.sciencedirect.com/topics/chemistry/quinine-sulphate) as the reference.

### 3.10 Lasing aspects

For power lasing properties, branching ratios (*β*_exp_) are desirable characteristics. The branching ratio value for ^4^G_5/2_ → ^6^H_7/2_ transition in the powder form is more than 50%, and the complexes have materiality in laser devices, whereas, in the solution phase, the branching ratio value for ^4^G_5/2_ → ^6^H_9/2_ transition possesses higher branching ratios than the ^4^G_5/2_ → ^6^H_7/2_ transition; hence, the former transition is applicable in lasing devices. In order to consider high-lasing behaviour, *β*_exp_ must be greater than 50%. The *β*_exp_ values are the ratio of integrated intensity of the corresponding peak to the total intensity of all peaks instigated from the ground state. Since *β*_exp_ of solid-state complexes was found to be close to 50% for the ^4^G_5/2_ → ^6^H_7/2_ transition, and for the solution phase, it is close to 50% for ^4^G_5/2_ → ^6^H_9/2_ transition (Table 8), so the lasing properties were obtained for the respective transitions in different phases. The stimulated emission cross-section (SEC) is a vital parameter to describe the utility of these complexes in laser devices, defence radar and telecommunication field. The SEC values of all complexes for the most prominent transition of emission spectra were evaluated using the following Fuchtbauer–Landenberg formula:14
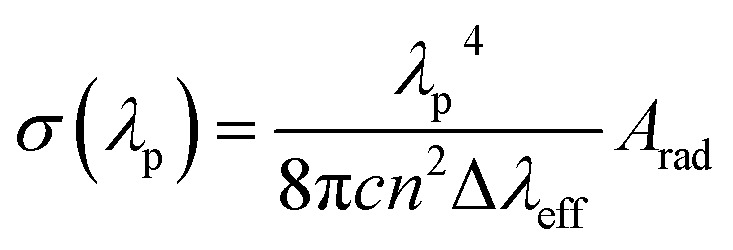
Here *λ*_p_ is the average emission peak, Δ*λ*_eff_ stands for the full width at half maxima (FWHM) for particular emission transition, *c* is the velocity of light and *n* is the refractive index. *A*_rad_ is the radiative transition probability of the most intense peak. FWHM is assessed for the most intense emission peak (^4^G_5/2_ → ^6^H_7/2_) for the solid phase and ^4^G_5/2_ → ^6^H_9/2_ transition for the solution of all complexes by applying the Gaussian fitting, as depicted in Fig. S12(a) and (b)† respectively. The optical parameters gain bandwidth (*σ*(*λ*_p_)*Δ*λ*_eff_ × 10^−28^ cm^3^) and optical gain (*σ*(*λ*_p_)*τ_R_ × 10^−25^ cm^2^ s) were also estimated using the SEC.^87,88^ Laser device amplification is dependent on the optical gain (*σ*(*λ*_p_)*τ_R_ × 10^−25^ cm^2^ s) and the values are listed in Table 8. The outcomes of all parameters of complexes (C1–C6) are comparable with erbium and samarium-doped glass value.^87–89^ The lasing properties of the synthesised complexes were compared with previously reported similar Sm^3+^ complexes (Table 8). In the C1–C6 complexes, gain bandwidth values are higher than the literature value, suggesting that the materiality of these complexes is in the broad band range optical devices. Further, the authors also determine the rarely reported, lasing properties in the solution phase of similar systems.

**Table 8:** Emission peak wavelength (*λ*_p_ nm), FWHM (Δ*λ*_eff_ nm), SEC(*σ*(*λ*_p_) × 10^−22^ cm^2^), gain bandwidth (*σ*(*λ*_p_) × Δ*λ*_eff_ × 10^−28^ cm^3^), optical gain (*σ*(*λ*_p_) × *τ*_R_ × 10^−25^ cm^2^ s) and *β*_exp_ (%) for the most intense emission transition ^4^G_5/2_ → ^6^H_7/2_ and ^4^G_5/2_ → ^6^H_9/2_ for solid and solution phases of C1–C6 complexes with some other reported complexes

Complexes		*λ*_p_	Δ*λ*_eff_	*σ*(*λ*_p_)	*σ*(*λ*_p_) × Δ*λ*_eff_	*β*_exp_ (%)	*σ*(*λ*_p_) × *τ*_R_	Reference
C1 (solid)	^4^G_5/2_ → ^6^H_7/2_	603.2	25.10	2.35	5.91	53.79	6.96	This work
C1 (solution)	^**4**^ **G**_**5/2**_ **→** ^**6**^ **H**_**9/2**_	649.2	10.93	7.26	7.94	50.12	21.47	
C2 (solid)	^4^G_5/2_ → ^6^H_7/2_	603.3	22.65	3.53	8.01	53.43	8.61	This work
C2 (solution)	^**4**^ **G**_**5/2**_ **→** ^**6**^ **H**_**9/2**_	649.0	10.41	10.33	10.75	50.67	25.13	
C3 (solid)	^4^G_5/2_ → ^6^H_7/2_	603.1	20.30	6.97	14.16	55.00	13.20	This work
C3 (solution)	^**4**^ **G**_**5/2**_ **→** ^**6**^ **H**_**9/2**_	649.3	10.11	18.80	19.00	51.23	35.57	
C4 (solid)	^4^G_5/2_ → ^6^H_7/2_	603.2	20.23	9.14	18.49	55.36	13.84	This work
C4 (solution)	^**4**^ **G**_**5/2**_ **→** ^**6**^ **H**_**9/2**_	649.1	10.10	24.56	24.81	51.17	37.20	
C5 (solid)	^4^G_5/2_ → ^6^H_7/2_	603.1	20.34	5.29	10.76	56.76	10.17	This work
C5 (solution)	^**4**^ **G**_**5/2**_ **→** ^**6**^ **H**_**9/2**_	649.5	9.93	14.54	14.44	51.48	27.95	
C6 (solid)	^4^G_5/2_ → ^6^H_7/2_	603.3	18.90	7.63	13.29	56.87	11.71	This work
C6 (solution)	^**4**^ **G**_**5/2**_ **→** ^**6**^ **H**_**9/2**_	649.3	9.83	18.15	17.84	51.90	30.22	
[Sm(L)_3_·(H_2_O)_2_]	^4^G_5/2_ → ^6^H_7/2_	606	15.58	2.12	3.31	—	5.10	74
[Sm(L)_3_·dmph]	^4^G_5/2_ → ^6^H_7/2_	606	15.91	2.73	4.34	—	4.99	74
[Sm(L)_3_·batho]	^4^G_5/2_ → ^6^H_7/2_	606	15.02	7.25	0.10	—	5.29	74
[Sm(L)_3_·neo]	^4^G_5/2_ → ^6^H_7/2_	606	14.86	1.66	2.41	—	5.35	74
[Sm(L)_3_·phen]	^4^G_5/2_ → ^6^H_7/2_	606	14.43	0.98	1.44	—	5.51	74
[Sm(L)_3_·bypy]	^4^G_5/2_ → ^6^H_7/2_	606	13.40	2.07	2.78	—	5.93	74
[Sm(L)_3_(H_2_O)_2_]·15H_2_O	^4^G_5/2_ → ^6^H_7/2_	604.4	23.01	0.103	—	—	—	5
[Sm(L)_3_dmph]·15H_2_O	^4^G_5/2_ → ^6^H_7/2_	603.5	22.93	0.104	—	—	—	5
[Sm(L)_3_bipy]·15H_2_O	^4^G_5/2_ → ^6^H_7/2_	603.8	22.73	0.104	—	—	—	5
[Sm(L)phen]·15H_2_O	^4^G_5/2_ → ^6^H_7/2_	605.5	22.42	0.107	—	—	—	5
[Sm(L)_3_]·15H_2_O	^4^G_5/2_ → ^6^H_7/2_	604.5	21.59	0.112	—	—	—	5

### 3.11 Colorimetric aspects

**Fig. 13:**
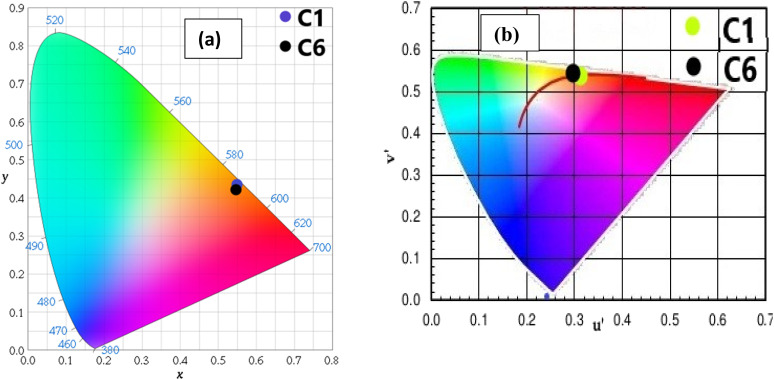
(a) CIE chromaticity coordinates of complexes C1 and C6 demarcated in the orange region of colour gamut space and (b) *u*′ and *v*′ coordinates of complexes C1 and C6 in the solid state on CCT chart showing their emission in warm orange light.

The CIE (Commission International de I′ Eclairage) color coordinate values of C1–C6 complexes were calculated using the MATLAB software with emission data in the solution as well as powder form. The position of color coordinates for all complexes was in orange gamut for the solid phase, as depicted in Fig. 13(a) and the reddish-orange gamut in the solution phase, as displayed in Fig. S13(a).† The observed color coordinates for powder forms are listed in Table 9, and for solution phase, are given in Table 10, which are harmonised with amber LED NSPAR 70BS (0.570, 0.420).^90^ Further, the positions of ternary complexes having secondary ligands are shifted in the brighter orange region relative to the binary complex due to the synergistic effect of secondary ligands.

**Table 9:** Photophysical parameters of C1–C6 complexes in solid state^a^

	*λ*_em_ (nm)	*I*_Sm_	*x*, *y*	CP (%)	CCT (K)	*u*′, *v*′	*τ* (ms)	*ϕ* (%)
C1	603	0.36	0.5592, 0.4398	94.27	1916.27	0.3124, .5528	1.30	43.98
C2	603	0.42	0.5652, 0.4338	94.98	1848.02	0.3195, 0.5518	1.40	57.53
C3	603	0.37	0.5644, 0.4346	95.36	1852.51	0.3185, 0.5519	1.71	80.84
C4	604	0.42	0.5540, 0.4409	96.61	1928.10	0.3085, 0.5524	2.40	84.78
C5	603	0.34	0.5561, 0.4403	98.14	1922.24	0.3101, 0.5525	2.53	88.03
C6	604	0.43	0.5560, 0.4409	98.45	1930.10	0.3098, 0.5527	2.88	90.09

a *λ*_em_ = emission wavelength (https://www.sciencedirect.com/topics/chemistry/emission-wavelength) for ^4^G_5/2_ → ^6^H_7/2_ transition; *I*_Sm_ = intensity ratio of electric dipole (^4^G_5/2_ → ^6^H_9/2_) to magnetic dipole transition (^4^G_5/2_ → ^6^H_5/2_); CP = color purity percentage; CCT = correlated color temperature; *u*′, *v*′ = coordinates for CCT; *τ* = decay time of luminescence (https://www.sciencedirect.com/topics/chemistry/luminescence-type); *ϕ* = intrinsic quantum yield (https://www.sciencedirect.com/topics/chemistry/quantum-yield) in solid state.

**Table 10:** Photoluminescence (https://www.sciencedirect.com/topics/chemistry/photoluminescence) data of C1–C6 complexes in the solution with DMSO as the solvent^a^

Complexes	*λ*_em_	*I*_Sm_	*x*, *y*	CP (%)	CCT (K)	*u*′,*v*′	*η* (%)
C1	603	6.93	0.5624, 0.3389	67.78	1705.94	0.3785, 0.5133	13.39
C2	603	7.13	0.5857, 0.3515	79.75	1725.56	0.3874, 0.5231	21.47
C3	603	7.17	0.5910, 0.3590	81.56	1698.40	0.3858, 0.5274	22.12
C4	604	6.57	0.6123, 0.3543	85.88	1735.99	0.4092, 0.5290	22.27
C5	603	7.12	0.6058, 0.3681	88.85	1725.53	0.3904, 0.5338	25.42
C6	604	10.1	0.6159, 0.3696	91.48	1736.83	0.4092, 0.5362	28.38

a *λ*_em_ = emission wavelength (https://www.sciencedirect.com/topics/chemistry/emission-wavelength) for ^4^G_5/2_ → ^6^H_7/2_ transition; *I*_Sm_ = intensity ratio of electric dipole (^4^G_5/2_ → ^6^H_9/2_) to magnetic dipole transition (^4^G_5/2_ → ^6^H_5/2_); CP = color purity percentage; *η* = relative quantum yield (https://www.sciencedirect.com/topics/chemistry/quantum-yield) with quinine sulphate (https://www.sciencedirect.com/topics/chemistry/quinine-sulphate) as reference.

Moreover, the percentage color purity (CP%) was assessed by the following relation to define the emitting performance of C1–C6 complexes in solid and solution phases. The color purity (CP) is an aspect to designate the purity of color produced by C1–C6 complexes, which was estimated using the following equation:^75,91^15
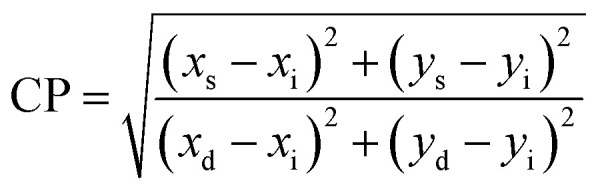
Here, the CP value was calculated concerning the white light color coordinate (*x*_i_, *y*_i_) and dominated color coordinate of complexes (*x*_d_, *y*_d_). Excellent CP% of all luminescent complexes was observed within the range of 92–98% in the solid phase and 67–91% in the solution phase, as mentioned in Tables 9 and 10 respectively. It is worthy to note that the color purity of the synthesised complexes is higher and better than that of the previously reported [Sm(ligand)_3_·secondary ligand] complexes in the literature, as given in Table 11.

**Table 11:** Comparison of the decay time and color purity of C1–C6 with other [Sm(ligand)_3_·secondary] complexes

Complex	Decay time (ms)	Color purity (CP) solid	Reference
Sm(L)_3_·H_2_O	1.30	94.27	This work
[Sm(L)_3_·bipy]	1.40	94.98	This work
[Sm(L)_3_·neo]	1.53	95.36	This work
[Sm(L)_3_·dmph]	1.63	96.61	This work
[Sm(L)_3_·batho]	1.33	98.14	This work
[Sm(L)_3_·phen]	1.50	98.45	This work
[Sm(L)_3_·(H_2_O)_2_]	0.764	86.83	5
[Sm(L)_3_·dmph]	1.071	87.13	5
[Sm(L)_3_·bipy]	1.27	86.90	5
Sm(L)_3_·_2_H_2_O	0.435	61.40	81
[Sm(L)_3_·neo]	0.757	72.65	81
[Sm(L)_3_·bipy]	0.891	63.40	81
[Sm(L)_3_·batho]	0.921	69.49	81
[Sm(L)_3_·phen]	1.510	76.15	81
[Sm(L)_3_·(H_2_O)_2_]	0.443	—	77
[Sm(L)_3_·Phen]	0.452	—	77
[Sm(L)_3_·Bipy]	0.495	—	77
[Sm(L)_3_·Neo]	0.546	—	77
[Sm(L)_3_·Batho]	0.550	—	77
Sm(TTA)_3_·Phen	0.043	—	82
Sm(TTA)_3_·PBr	0.039	—	82
Sm(TTA)_3_·MP	0.027	—	82
Sm(TTA)_3_DP	0.018	—	82
[Sm(MAE)_3_·2H_2_O]	0.57	42.24	83
[Sm(MAE)_3_·(dmbipy)]	1.18	47.37	83
[Sm(MAE)_3_·(bipy)]	1.25	64.48	83
[Sm(MAE)_3_·(batho)]	1.42	73.63	83
[Sm(MAE)_3_·(phen)]	1.58	78.96	83

The CCT values are utilised to avoid visual and mental difficulties. To further envision the quality of emitted light by luminescent complexes, the CCT (correlated color temperature), was obtained by the Mc-Camy method:^92^

16 CCT = −437*n*^3^ + 3601*n*^2^ − 6861*n* + 5514.31

The evaluation of n can be done as *n* = (*x* − *x*_e_)/(*y* − *y*_e_).

Other key aspects such as *u*′ and *v*′ were explored using the following expression:17
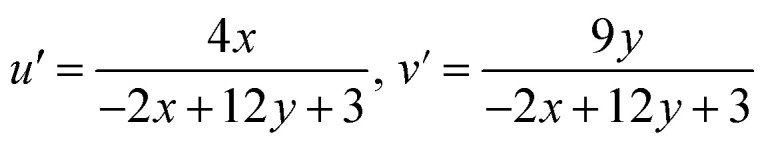


The luminescent light sources can be cool, warm or neutral at a temperature above 4000 K, below 3200 K and between 3200 and 4000 K respectively, which could be decided by the outcomes of their CCT and their *u*′ and *v*′ coordinate values. The values of *u*′ and *v*′ and CCT for the synthesized complexes recommended that the complexes are valuable candidates for the successful encapsulation in warm orange and reddish-orange light-emitting sources such as home appliances and living rooms.^86^Tables 9 and 10 list the data of CCT and *u*′ and *v*′ outcomes for solid and solution states respectively. Through the (*u*′,*v*′) coordinates, the CCT value of complexes is presented in the CCT chart; the value in the solid state is displayed in Fig. 13(b) and that in the solution state in Fig. S13(b).† However, the observed CCT values are consistent with other Sm(iii) complexes existing in the literature.^77,93^ The Judd–Ofelt values, decay time, color purity, intrinsic quantum efficiency and relative quantum efficiency values of previously reported similar systems are compared and presented in Table S2 in the ESI.† The outcomes of the as-synthesised complexes are comparable with the similar systems.

### 3.12 Energy transfer

**Fig. 14:**
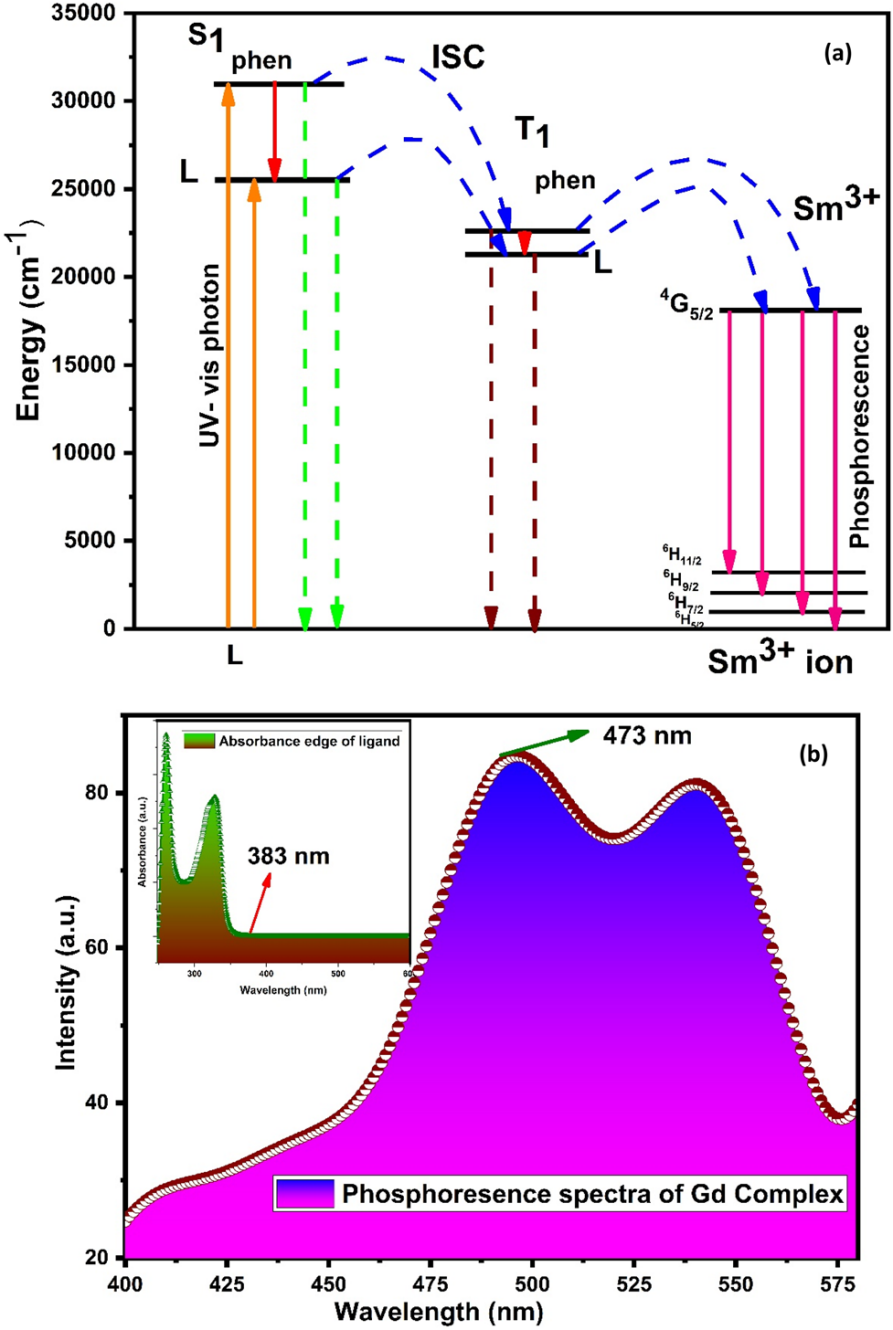
(a) Schematic energy level and proposed energy transfer mechanism of the C6 complex and (b) phosphorescence spectra of [Gd(L)_3_·2H_2_O]·3H_2_O; inset illustrates the absorption spectra of L.

The main effect on the sensitization process of Sm^3+^ ions by L could be understood by the energy transfer mechanism. Transfer mechanism was based on the difference in energy (Δ*E*) between the triplet state (T_1_) of L and the emissive level of Sm^3+^ ions and the life time of the triplet state. For the evaluation of energies for excited singlet state (S_1_) and excited triplet state (T_1_) of L, a binary gadolinium(iii) complex was prepared by a similar method, which was applied for the synthesis of a binary samarium(iii) complex. The excited singlet (S_1_) energy values of L and secondary ligands were calculated from their edge wavelengths of absorption spectra. The inverse of edge wavelength gives the energy of S_1_ in cm^−1^ and these values are documented in Table 12. The excited triplet (T_1_) level of energy was estimated by the shortest emission wavelength of phosphorescence spectra of gadolinium(iii) complexes of L and secondary ligands. In case of Gd^3+^ complexes, the energy transfer from L to metal ions is less feasible because the emitting level of Gd^3+^ ions is located very high at 32 000 cm^−1^ compared to the triplet excited state of L.^94,95^ The inverse of the shortest emission wavelength is the value T_1_ in cm^−1^ and these values are documented in Table 12. The absorption edge wavelength and shortest emission wavelength of phosphorescence spectra of Gd^3+^ complexes of L are displayed in Fig. 14(b).

**Table 12:** Energies of L, phen, bipy, dmph, neo and batho ancillary ligands

Ligands	Energy levels		Δ*E*_(S_1_ → T_1_)_ (cm^−1^)	Δ*E*_(T_1_ → ^4^G_5/2_)_ (cm^−1^)
	Singlet (cm^−1^)	Triplet (cm^−1^)		
L	26 109	21 141	4968	3284
Phen	31 000	22 100	8900	4243
Bipy	29 900	22 900	7000	5043
Dmph	31 250	21 097	10 154	3240
Neo	30 750	22 624	8126	4667
Bathophen	29 000	21 000	8000	3143

Fig. S14† shows the overlapping of excitation spectra of complex C1 and absorption spectra of L, which reveal the better coordination of antenna ligands with Sm^3+^ ions and the more efficient sensitization process by L. The energy transfer pathway [S_0_ → S_1_ → T_1_ → emissive level of Sm^3+^ ion (^4^G_5/2_) → radiative transition relaxed in the ground (^6^H_5/2, 7/2, 9/2, 11/2_)]^5^ is illustrated in Fig. 14(a). Two principles have affected the efficiency of energy transfer to metal ions by the ligand: one is thermal deactivation theory (inverse energy transfer) and the other is Dexter's resonance exchange theory (suitable energy difference Δ*E*_T_1_ → ^4^G_5/2__). Similarly, according to Latva's empirical rule, the values of Δ*E*_T_1_ → ^4^G_5/2__ within 2000–5000 cm^−1^ range are imperative for effective energy transfer. The Δ*E*_T_1_ → ^4^G_5/2__ value of L was 3284 cm^−1^ that was optimum for the sensitisation of Sm^3+^ ions *via* organic L.^77^

### 3.13 Computational studies

**Fig. 15:**
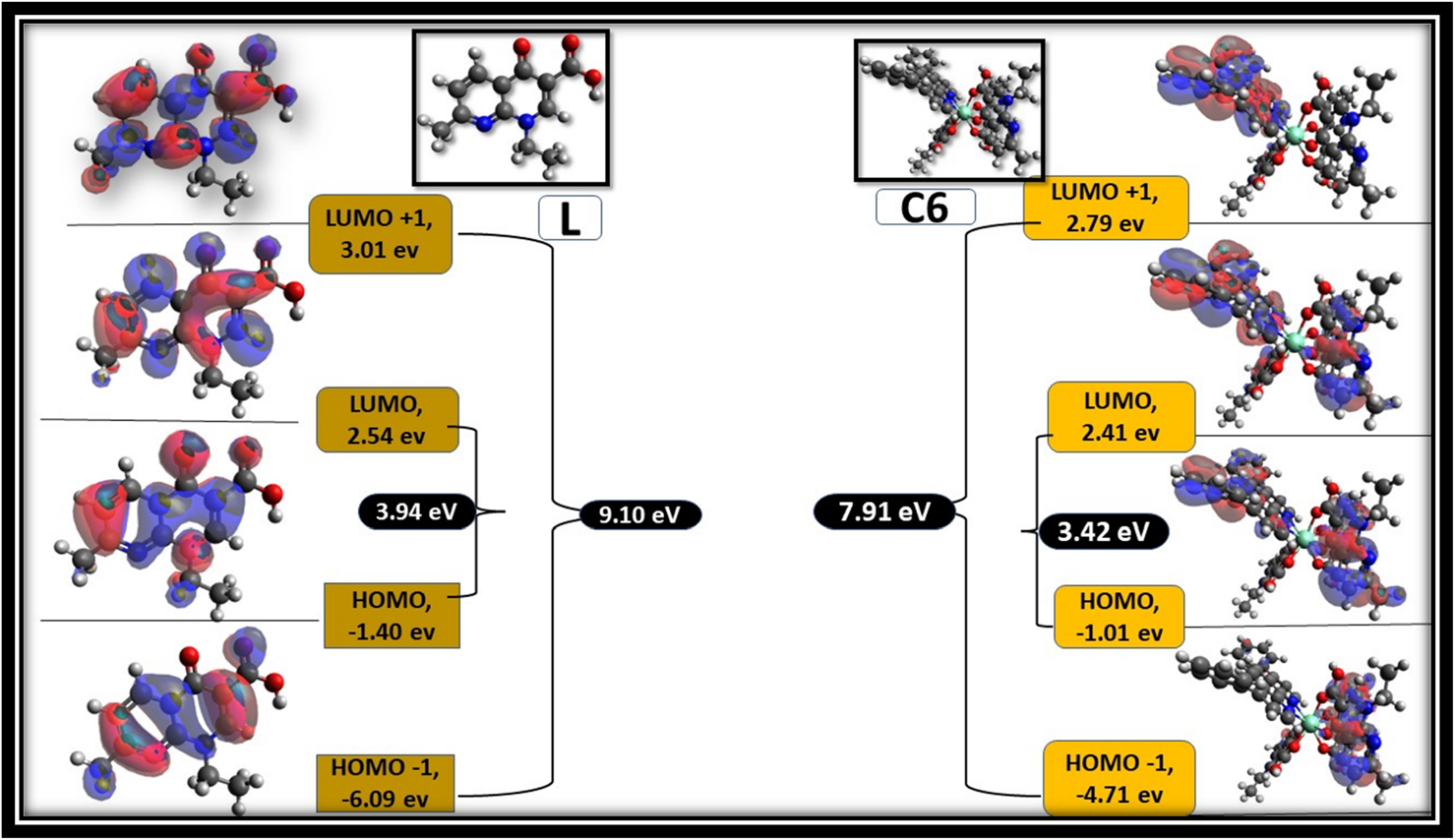
Frontier molecular orbitals of L and C6 complexes with their respective values.

Avogadro software is an auto-optimisation tool, which is used to build a molecule with minimised energy.^96^ The minimised energy molecule in the Avogadro software was used to generate an ORCA input file for the DFT calculation using the ORCA software.^97^ Single-point energy calculations were performed at the B3LYP level of density functional theory (DFT) with basis set def2-SVP.^83^ Frontier molecular orbitals (FMOs) were visualised using the Avogadro software from the ORCA output file. Fig. 15 shows the selected FMOs (LUMO+1, LUMO, HOMO and HOMO−1) and optimised structure of free L and C6 complexes with the HOMO–LUMO energy gap. Table S3† systemised the FMOs with their respective energies of the C1–C5 complexes in the ESI.† The development of FMOs is due to orbitals originating from the L and secondary ligands rather than the metal ion. The probability distribution of frontier orbitals suggests that the antibonding orbital of ligands such as LUMO and LUMO+1 are dominantly restricted on the secondary ligands, while the bonding molecular orbitals such as HOMO and HOMO−1 are confined on L.^95,98^ From the DFT calculation, the optical band gap values of ligand and C1–C6 complexes were estimated by the difference between HOMO and LUMO orbitals. The optical band gap value of the ligand is 3.98 eV, and that of complexes lies in 3.20–3.44 eV range, suggesting that the energy gap was decreased in complexes, which is in accordance with the experimental values obtained from the absorbance data.^99^

Tjalling Koopmans state that the HOMO and LUMO orbitals are helpful in the estimation of electron affinity (*A*) and ionisation energy (*I*) of the synthesised complexes, as given in the following relation:^100^

18 *A* = −*E*_LUMO_, *I* = −*E*_HOMO_

Chemical softness (*σ*), electronegativity (*χ*), hardness (*η*) and potential (*μ*) are some other parameters calculated using *A* and *I* outcomes.^101^ All outcomes' values are documented in Table 13.19
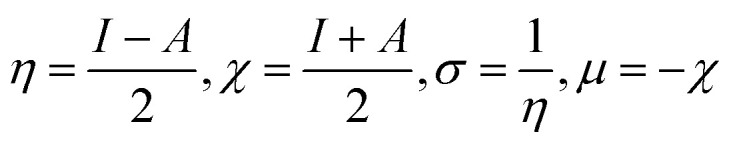


**Table 13:** IC_50_ values for the antioxidant activities of L and all C1–C6 complexes

Complexes	Concentration (μg mL^−1^)				
	25	50	75	100	IC_50_
L	32.22	42.09	49.89	56.57	76.62
C1	34.01	44.44	52.18	62.09	67.43
C2	37.68	45.64	53.13	60.04	65.43
C3	36.76	43.95	54.19	59.78	66.64
C4	41.76	50.73	55.98	63.09	52.09
C5	42.97	48.09	57.18	66.14	51.23
C6	40.53	50.75	57.84	67.19	50.84
STD	45.18	52.12	60.18	68.32	41.70

### 3.14 Evaluation of biological properties

#### 3.14.1 Antioxidant features

**Fig. 16:**
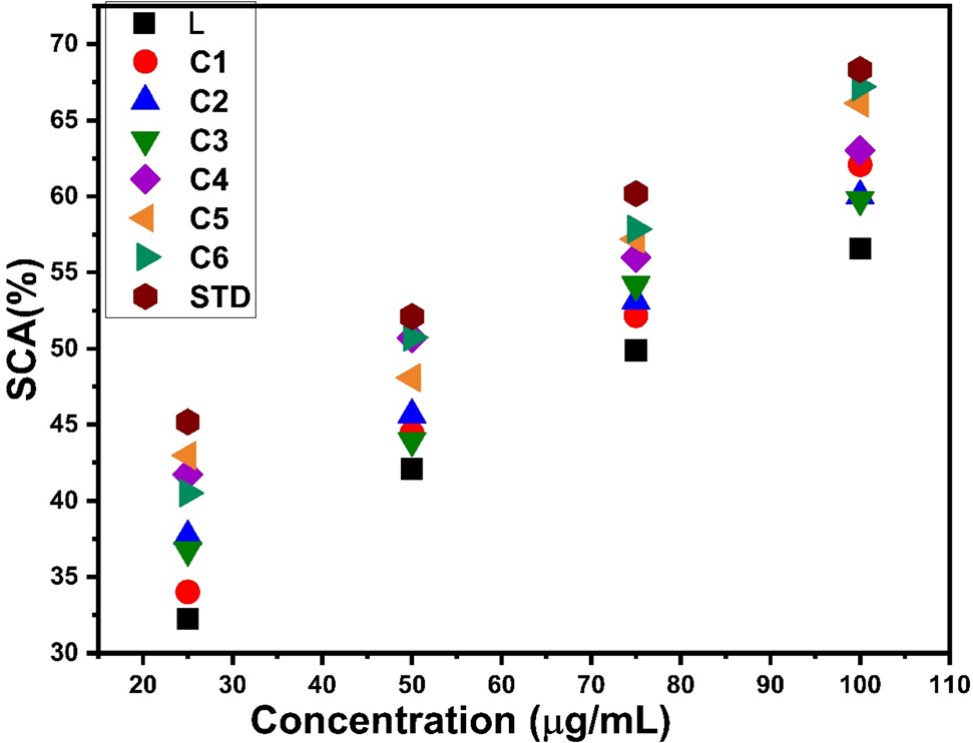
Percentage scavenging activity of C1–C6 and ligand with respect to the standard.

The anti-oxidant activity test of all complexes was conducted using DPPH as the free radical and ascorbic acid as the standard. It is noteworthy that in complexes C1–C6, a decrease in absorbance at 517 nm due to reduction of DPPH is observed. This reduction of DPPH can be explained by accepting protons from the complexes and stabilizing themselves. The antioxidant potential was expressed as IC_50_ values, which are listed in Table 13. The IC_50_ values were determined from the graph between different concentrations of samples and the percentage of scavenging activity of complexes exhibited in Fig. 16. All complexes C1–C6 show delocalization of π electrons, which is responsible for diminishing the absorbance and enhancing the scavenging activity of complexes.^102^ The IC_50_ value of all complexes is in the range of 50–70 μg mL^−1^, indicating more antioxidant activities than those of free L (76.62 μg mL^−1^), but less compared to STD (41.70 μg mL^−1^). The introduction of an additional chromophore moiety, a secondary ligand in C2–C6 complexes, further increases the antioxidant properties of complexes. Fig. S15† shows the comparison of IC_50_ values for L and synthesized complexes C1–C6 with respect to the standard drug.

#### 3.14.2 Antimicrobial activity

**Fig. 17:**
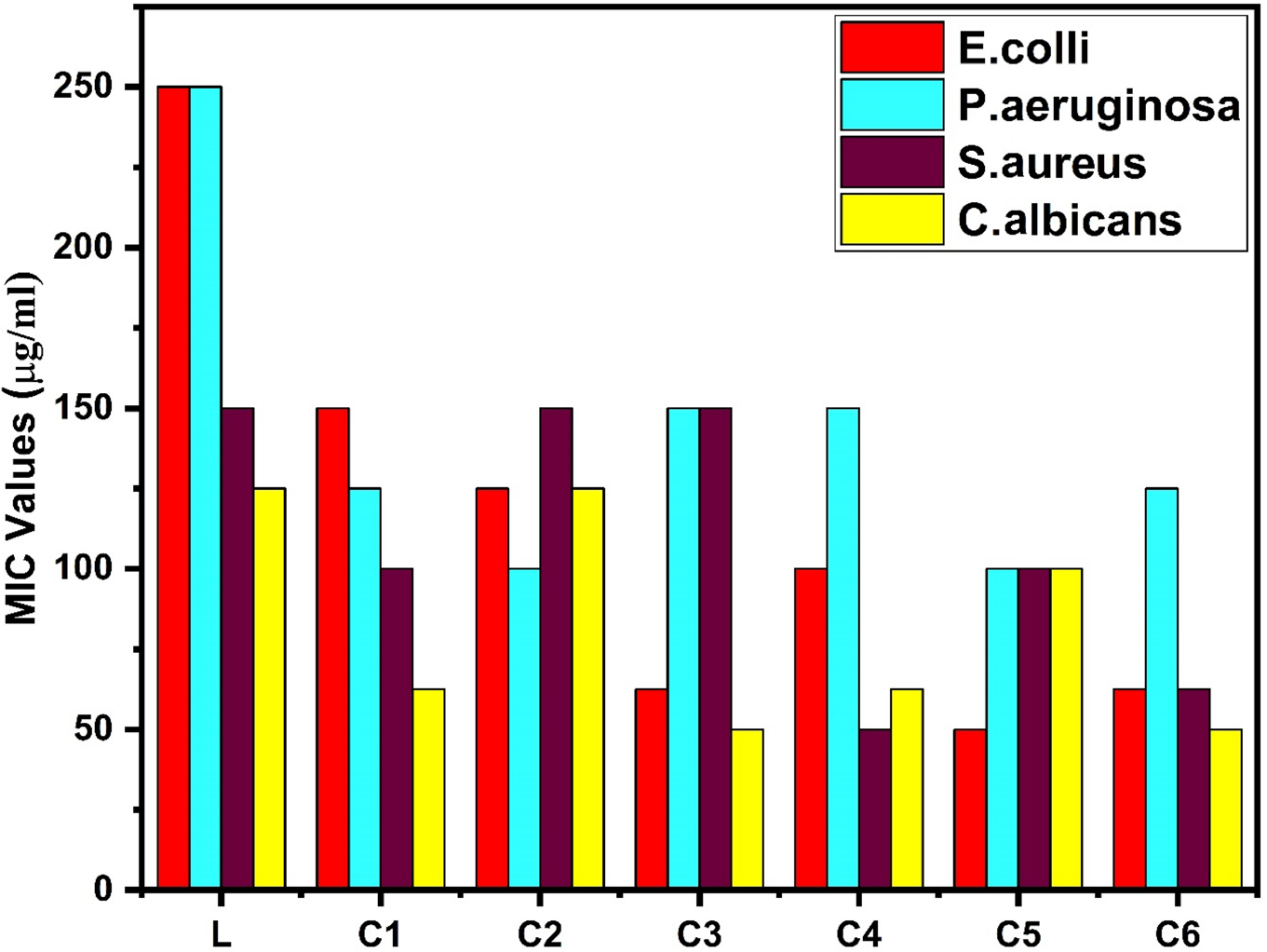
Antibacterial activity of L and C1–C6 in terms of MIC.

The antibacterial properties of all the synthesized complexes were tested against two Gram-positive and two-Gram negative bacterial strains. The MIC data of complexes are presented in Table 14 and the bar graph in Fig. 17, which represent the higher antibacterial activity of all the synthesized complexes than that of the free ligand, specifically against Gram-positive bacteria.^103^ Gram-negative bacteria have a protective lipid layer under the cell wall, which hinders the penetration of drugs to the cell.^104^ Samarium(iii) complexes show better antibacterial activity against *Staphylococcus aureus* among all bacteria. The increased activity of complexes could be easily explained by Overtone's theory and Tweedy's chelation theory.^105,106^ The polarity of the ligand was reduced by partial sharing of the +ve charge of the metal with the donor (ligand). In the process of complexation, diminishment of polarity may increase lipophilicity, and the lipophilic behaviour of complexes favours the penetration and transportation in the lipid membrane of the cell. Moreover, it disturbs the respiration process and blocks protein synthesis, which hampers the growth of organism.^107^ π electron delocalization over the ligand affected the lipid attraction ability of the ligand towards the metal ion. The existence of secondary ligands in C2–C6 complexes further enhances the delocalization of π electrons, which also increases the anti-microbial activity of complexes as compared to the C1 complex.

**Table 14:** Antimicrobial activities of L and all C1–C6 complexes

Compounds	Antibacterial activities in terms of their MIC values				Antifungal activities in terms of their MIC values		
	*E. coli*	*P. aeruginosa*	*S. aureus*	*S. pyogenes*	*C. albicans*	*A. niger*	*A. clavatus*
L	150	250	150	125	500	1000	1000
C1	150	125	100	62.5	500	500	500
C2	125	100	150	125	250	1000	500
C3	62.5	150	150	50	100	500	250
C4	100	150	50	62.5	250	500	1000
C5	50	100	100	100	100	250	250
C6	62.5	125	62.5	50	250	500	250
STD	—	—	—	—	500	100	100

The antifungal activities of samarium(iii) complexes and pure ligand were determined against three fungal strains. On comparing the MIC values of the pure ligand and complexes, interesting results were found, which show that the complexes have more antifungal activities than those of the ligand. All complexes show comparable activity against *Aspergillus clavatus* but excellent antifungal activity against *Candida albicans*. The antibacterial and antifungal activities of all complexes are displayed in Table 14, and hence, undoubtedly, these complexes can be used as antifungal agents and germ destroying agents.

## 4 Conclusions

The synthesis of six complexes, namely, C1–C6 with β-keto carboxylic acid and nitrogen-containing secondary ligands by a green liquid-assisted grinding method was reported in the present article. The characterisation of these synthesized complexes has been done precisely *via* NMR spectra, IR spectra, UV-vis spectra, thermal analysis, reflectance spectra, photoluminescence spectra, luminescence decay time, *etc.* These characterisation outcomes suggested the binding site of organic ligands, thermal stability and band gap energy values of complexes successfully. PL analysis of complexes was performed in a solution as well as a solid phase. The intense emissions assigned to the ^4^G_5/2_ → ^6^H_7/2_ transition in the solid phase and the ^4^G_5/2_ → ^6^H_9/2_ transition in the solution phase are creditworthy for orange and reddish orange color rendered in the UV-vis wavelength respectively. Urbach energy investigation was helpful to explain the applicability of these complexes in solar cells. Colorimetric features and antimicrobial properties have been evaluated effectively. The synthesised complexes can act as good anti-oxidant and anti-microbial agents in the pharmaceutical industry. Among all complexes, the C6 complex acts as the best antimicrobial agent against all bacterial strains. Our investigation outcomes reveal that the synthesized complexes are applicable as valuable candidates for numerous advanced photonic, medical and optical applications such as lightening systems, warm orange light sources, semiconducting materials and solar cells.

## Data availability

All data analysed during this study are included in this article and its ESI.†

## Author contributions

Poonam Kumari: investigation, conceptualisation, data curation, formal analysis, writing – original draft. Vaishnavi Lather: contributed in study of microbial properties. Savita Khatri: software, validation. Harkesh Sehrawat: computational analysis. Pratibha Ahlawat: writing – review and editing. S. P. Khatkar: writing – review and editing, visualisation, supervision. V. B. Taxak: writing – review and editing, visualisation, supervision. Rajesh Kumar: supervision, formal analysis, writing – review and editing.

## Conflicts of interest

The authors say no conflicts of interest regarding this research work.

## Supplementary Material

RA-012-D2RA05796D-s001
