# Supplementary material for: Computational analysis, Urbach energy and Judd–Ofelt parameter of warm Sm3+ complexes having applications in photovoltaic and display devices
Source: RSC Adv. 2022 Dec 15;12(55):35827–48. doi: 10.1039/d2ra05796d (PMC9753104; doi:10.1039/d2ra05796d)
Supplement: RA-012-D2RA05796D-s001 [file RA-012-D2RA05796D-s001.pdf]

Electronic Supplementary Material (ESI) for RSC Advances.
This journal is © The Royal Society of Chemistry 2022

Computational analysis, Urbach energy and Judd-Ofelt parameter of warm Sm^{3+} complexes possessing application in photovoltaic and display devices

Poonam Kumari[a], Vaishnavi Lather[c], Savita Khatri[a], Pratibha Ahlawat[a], Harkesh Sehrawat[a], S. P. Khatkar[b], V. B. Taxak[b] and Rajesh Kumar[a]*

[a]University Institute of Engineering and Technology, Maharshi Dayanand University, Rohtak - 124001, India

[b]Department of Chemistry, Maharshi Dayanand University, Rohtak - 124001, India

[c]Shri Guru Ram Rai Institute of medical and Health Sciences, Dehradun - 248001, India

*Corresponding author: Tel.: 9034070027

E-mail address: lather_rajesh@yahoo. com (Rajesh Kumar)

$$\%\,age\ inhibition = \frac{A_C - A_t}{A_C} \times 100 \quad \text{(S1)}$$

In the aforementioned equation A_c and A_t represents the absorbance of control (DPPH radical) and test samples respectively.

$$f^{exp} \leftrightarrow f^{cal} = \frac{8\pi^2 mc}{3h\lambda(2J+1)n^2}\frac{n(n^2+2)^2}{9}\left[\left(\Omega_2\left| < \psi_J \parallel U^2 \parallel \psi > \right|^2\right) + \left(\Omega_4\left| < \psi_J \parallel U^4\, \psi > \right|^2\right) + \left(\Omega_6\left| < \psi_J \parallel U^6 \parallel \psi > \right|^2\right)\right]$$
(S2)

$$A_{rad} = \frac{64e^2\pi^4}{3h(2J+1)\lambda^3}\frac{n(n^2+2)^2}{9} S_{Ed} \quad \text{(S3)}$$

Hare, S_{Ed} is the product of intensity parameter and reduced square matrix element.

$$\alpha = CF\,(R_\infty) \quad \text{(S4)}$$

$$F(R_\infty) = \frac{(1-R_\infty)^2}{2R_\infty} \quad \text{(S5)}$$

In aforementioned equation *C* is the constant and α is the absorption coefficient as Kubleka-Munk's function($F(R_\infty)$).

$$A_{total} = 1/\tau_{exp} = (A_{rad} + A_{nrad}) \quad \text{(S6)}$$

Table: S1 Molecular parameters of L and C1-C6 complexes.

Complexes	Chemical softness (σ)	Chemical electronegativity (χ),	Chemical Hardness (η)	Chemical potential (μ)
**L**	0.50	-0.57	1.97	0.57
**C1**	0.59	-0.53	1.69	0.53
**C2**	0.61	-0.39	1.63	0.39
**C3**	0.60	-0.53	1.66	0.53
**C4**	0.63	-0.14	1.57	0.14
**C5**	0.61	-0.55	1.62	0.55
**C6**	0.58	-0.70	1.71	0.70

Table S2. Comparison of Judd Ofelt properties, decay time, color purity, intrinsic quantum yield and relative quantum yield with other similar system given in literature.

Complexes	Ω_2 $\times 10^{-2}$	Ω_4 $\times 10$[illegible]	Ω_6 $\times 10^{-2}$	Trend	τ_{exp} (ms)	CP(%)	Φ (%)	η %	
$[Sm(L)_3.(H_2O)_2]$	15.53	1.32	3.70	$\Omega_2 > \Omega_6 > \Omega_4$	1.30	94.27	43.98	35.88	Present work
$[Sm(L)_3.bipy]$	17.88	1.96	4.88	$\Omega_2 > \Omega_6 > \Omega_4$	1.40	94.98	57.53	53.47	Present work
$[Sm(L)_3.dmph]$	18.36	3.41	8.82	$\Omega_2 > \Omega_6 > \Omega_4$	1.53	95.36	80.84	53.68	Present work
$[Sm(L)_3.batho]$	22.53	4.54	11.45	$\Omega_2 > \Omega_6 > \Omega_4$	1.63	96.61	84.78	59.31	Present work
$[Sm(L)_3.neo]$	22.58	1.44	7.72	$\Omega_2 > \Omega_6 > \Omega_4$	1.33	98.14	88.03	55.03	Present work

[Sm(L)$_3$.phen]	23.75	4.54	6.92	$\Omega_4 > \Omega_6 > \Omega_2$	1.50	98.45	90.09	67.27	Present work
[Sm(L)$_3$.(H$_2$O)$_2$]	14.26	1.14	3.02	$\Omega_2 > \Omega_6 > \Omega_4$	0.83	92.90	25.03	-	[1]
[Sm(L)$_3$.bipy]	19.35	0.17	1.54	$\Omega_2 > \Omega_6 > \Omega_4$	1.12	92.95	37.20	-	[1]
[Sm(L)$_3$.dmph]	21.58	0.35	2.04	$\Omega_2 > \Omega_6 > \Omega_4$	1.04	96.50	39.54	-	[1]
[Sm(L)$_3$.batho]	22.99	0.36	2.53	$\Omega_2 > \Omega_6 > \Omega_4$	1.41	96.47	58.54	-	[1]
[Sm(L)$_3$.neo]	22.78	0.50	2.52	$\Omega_2 > \Omega_6 > \Omega_4$	1.44	96.47	59.01	-	[1]
[Sm(L)$_3$.phen]	24.38	0.13	2.57	$\Omega_4 > \Omega_6 > \Omega_2$	1.74	97.84	74.35	-	[1]
[Sm(L)$_3$.(H$_2$O)$_2$]	4.44	0.55	2.28	$\Omega_2 > \Omega_6 > \Omega_4$	1.34	24.40	16.34	-	[2]
[Sm(L)$_3$.dmph]	7.71	0.74	2.92	$\Omega_2 > \Omega_6 > \Omega_4$	1.80	25.86	33.88	-	[2]
[Sm(L)$_3$.batho]	9.59	0.15	0.98	$\Omega_2 > \Omega_6 > \Omega_4$	1.92	25.86	32.64	-	[2]
[Sm(L)$_3$.neo]	10.71	0.27	1.70	$\Omega_2 > \Omega_6 > \Omega_4$	2.12	26.79	43.03	-	[2]
[Sm(L)$_3$.phen]	8.91	2.87	6.56	$\Omega_2 > \Omega_6 > \Omega_4$	2.34	27.42	25.28	-	[2]
[Sm(L)$_3$.bypy]	15.48	0.23	2.01	$\Omega_4 > \Omega_6 > \Omega_2$	2.96	34.87	83.28	-	[2]

Table: S3 Frontier Molecular Orbitals of C1-C5 complexes with their respective value.

**Complexes**	**C1**	**C2**	**C3**	**C4**	**C5**
	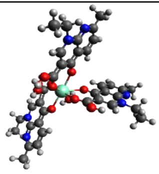	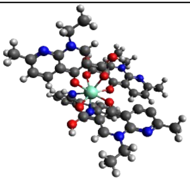	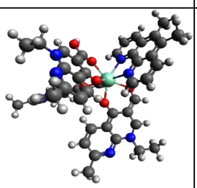	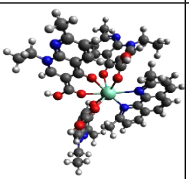	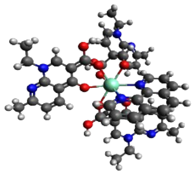

**LUMO +1**	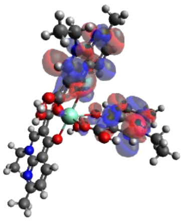	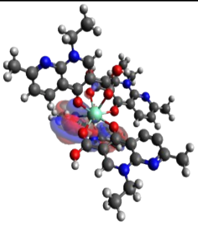	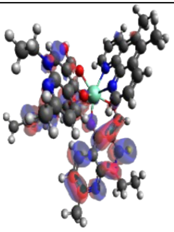	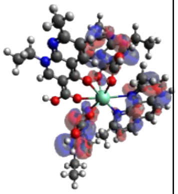	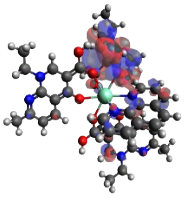
**Energy**	2.59 eV	2.53 eV	2.79 eV	1.97 eV	2.98 eV
**LUMO**	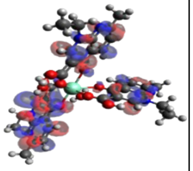	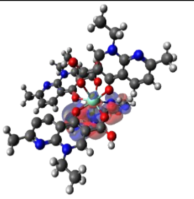	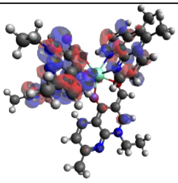	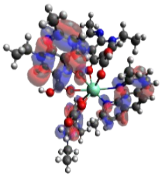	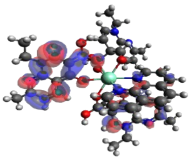
**Energy**	2.23 eV	2.02 eV	2.19 eV	1.43 eV	2.18 eV
**HOMO**	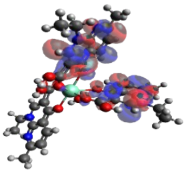	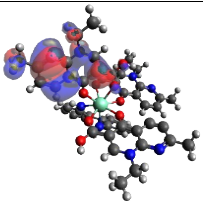	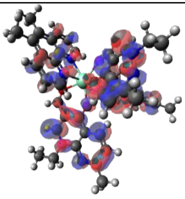	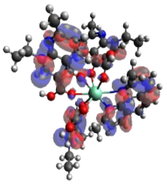	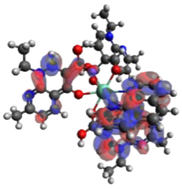
**Energy**	-1.16 eV	-1.24 eV	-1.13 eV	-1.71 eV	-1.07 eV
**HOMO -1**	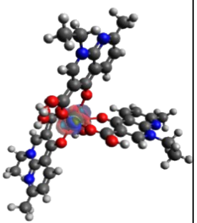	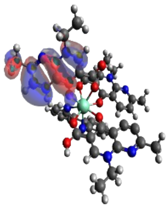	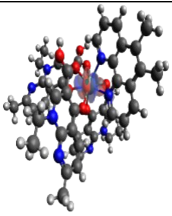	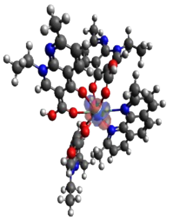	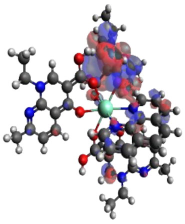
**Energy**	-5.27 eV	-4.98 eV	-5.03 eV	-5.60 eV	-5.04 eV

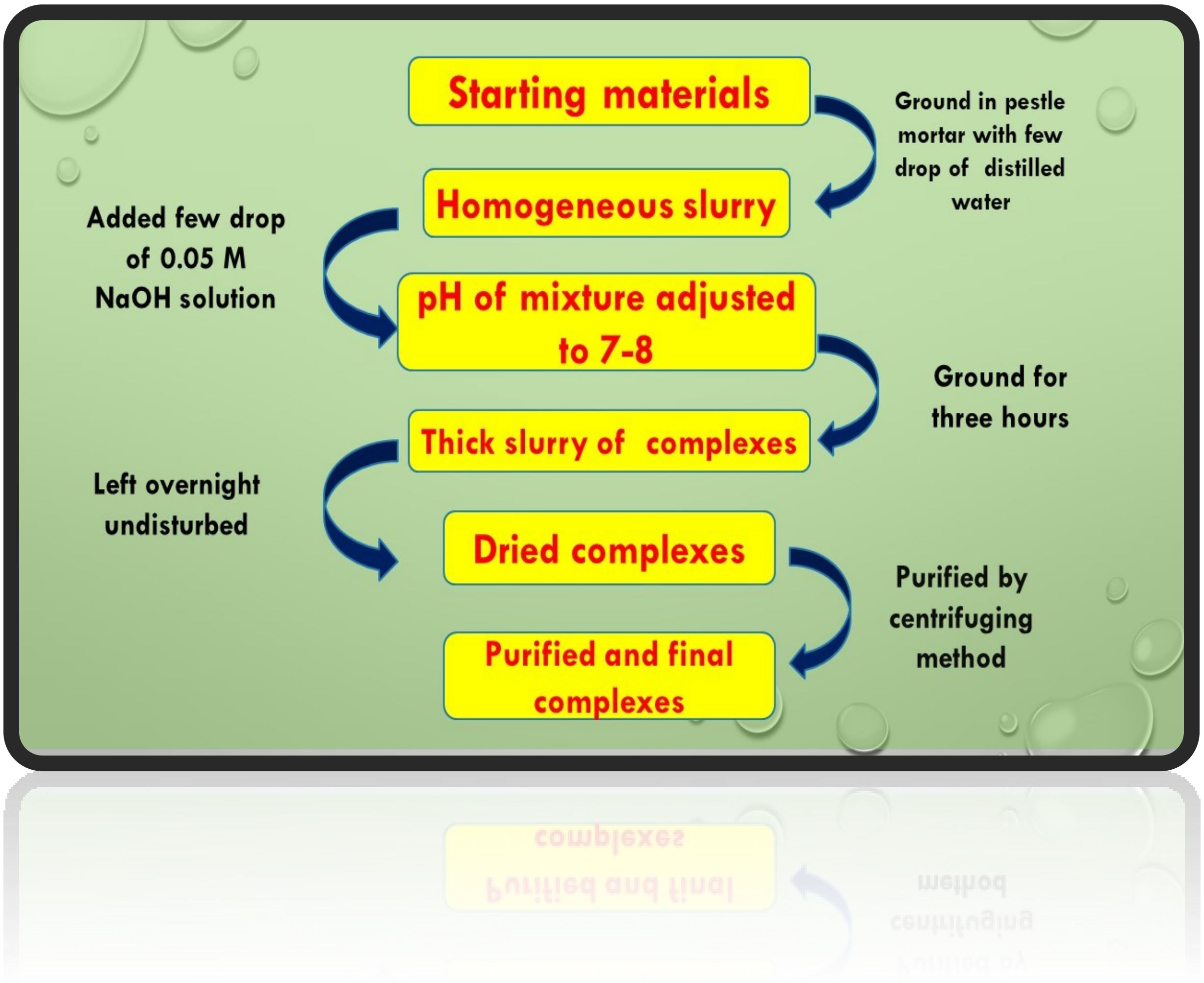

Fig. S1 Flowchart representing the steps of synthesis complexes by liquid assisted grinding method.

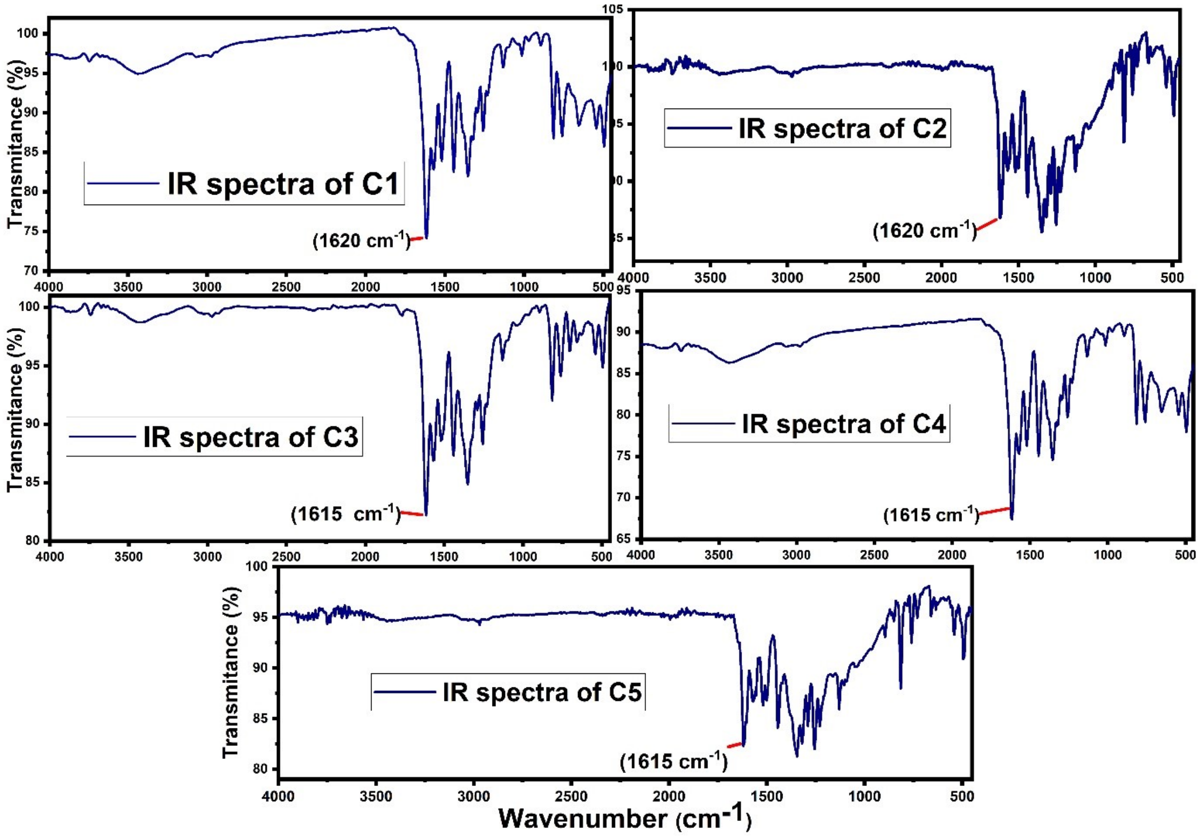

Fig. S2 FT-IR spectra of C1-C5 complexes in solid state.

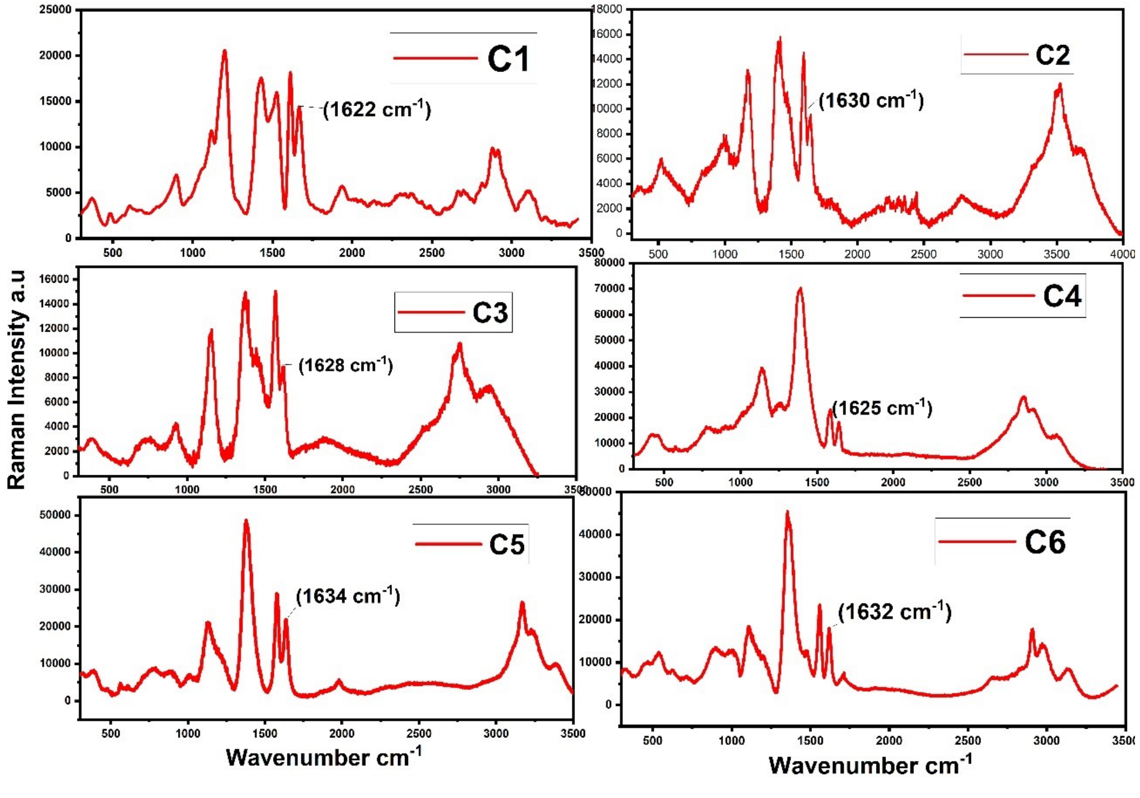

Fig. S3 Raman spectra of all complexes C1-C6 .

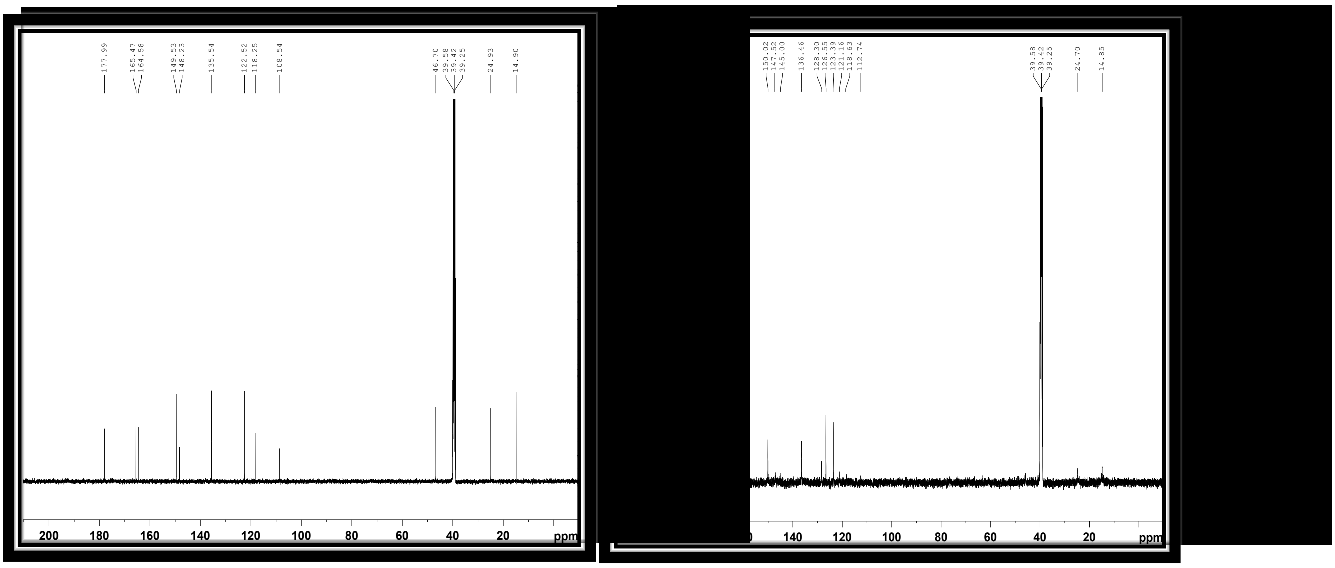

(a) **(b)**

Fig. S4(a) ^{13}C-NMR spectra of ligand and (b) ^{13}C-NMR spectra of C6 complex

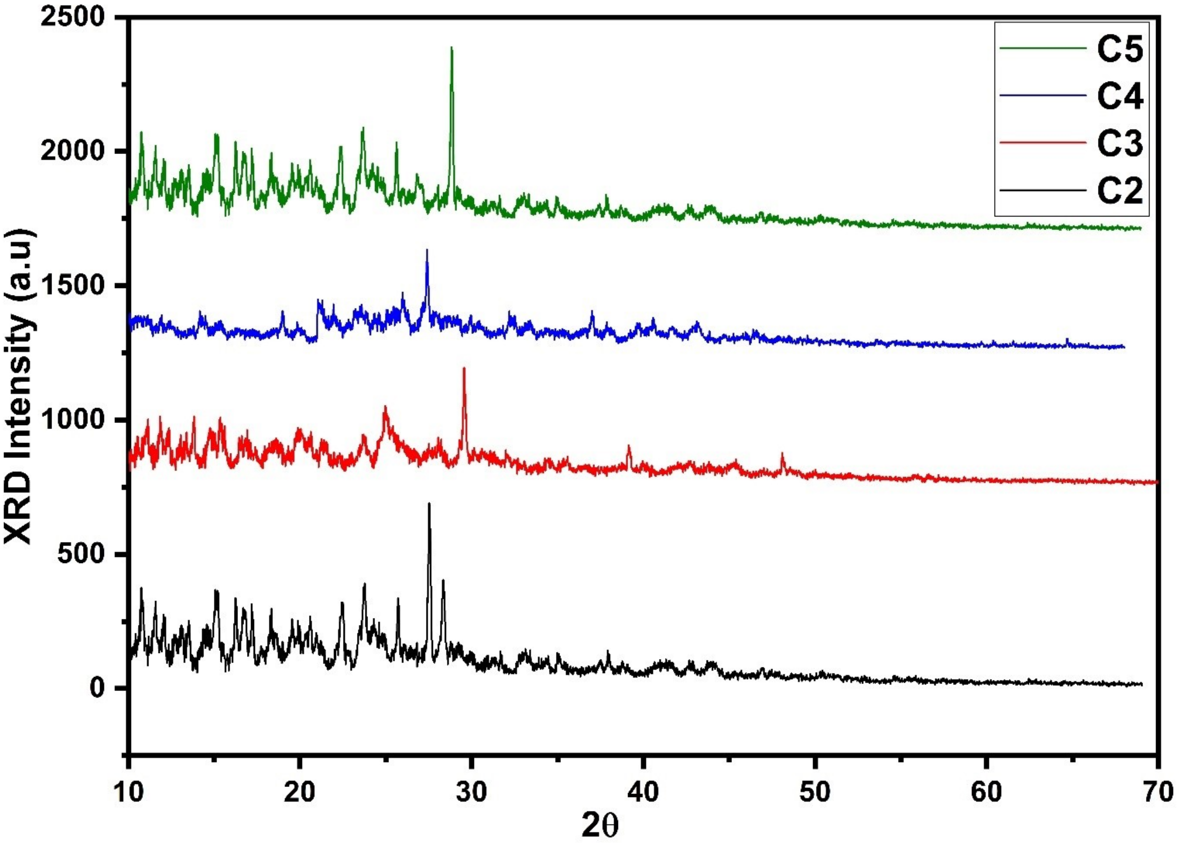

Fig. S5 PXRD patteran of C2-C5 complexes.

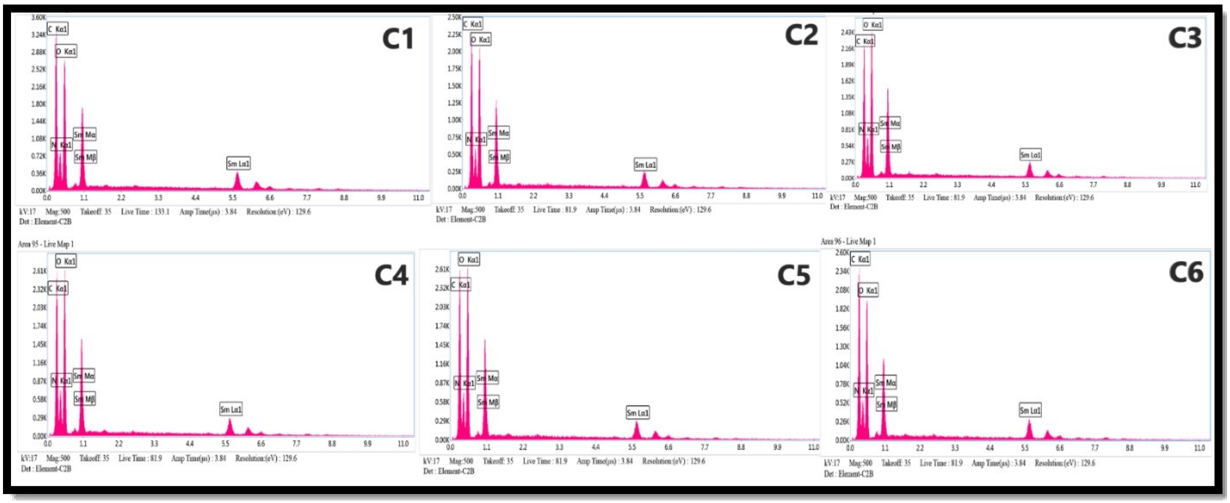

Fig. S6(a) EDAX spectra exhibiting compositional element of all complexes C1-C6.

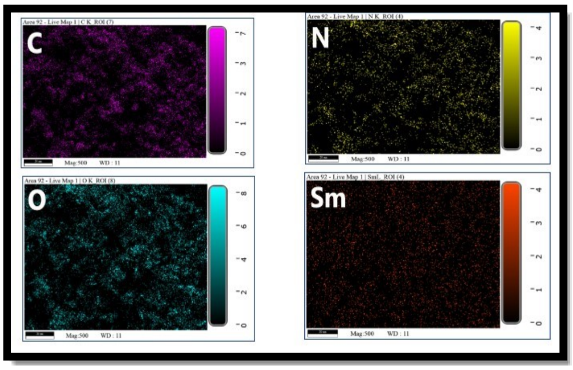

C1

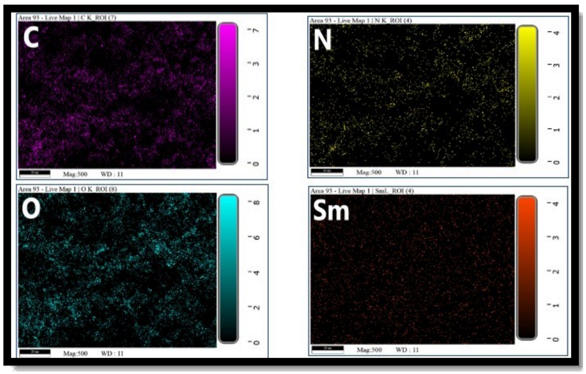

C2

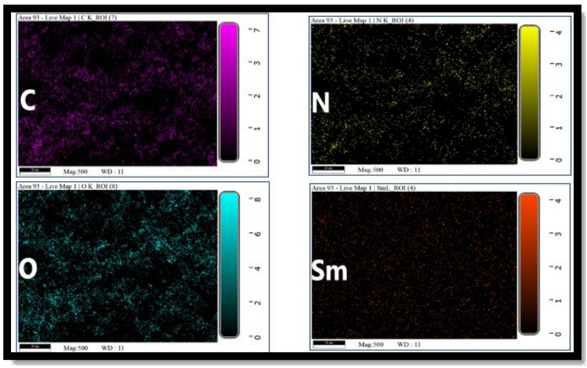

C3

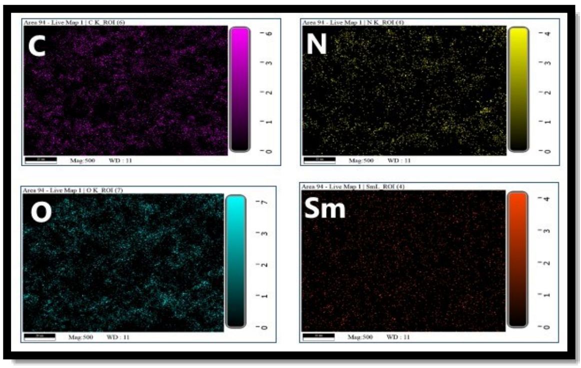

C4

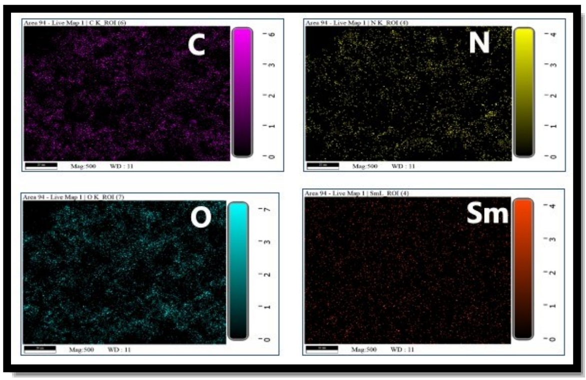

C5

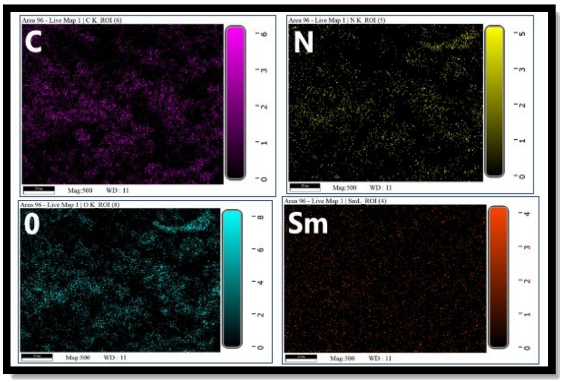

C6

Fig. S6(b) EDAX mapping of all C1-C6 complexes.

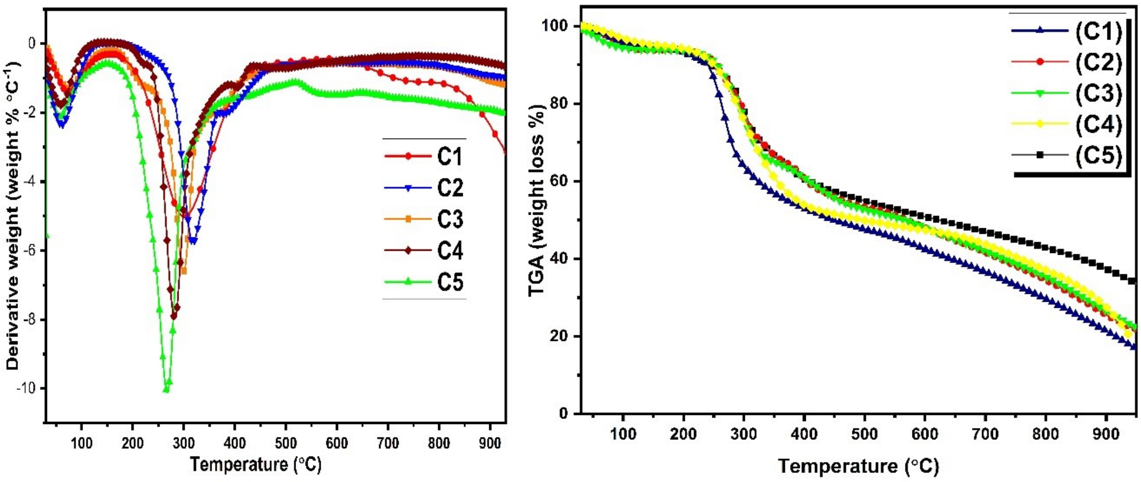

Fig. S7 TGA/DTG thermograph of C1-C5 complexes

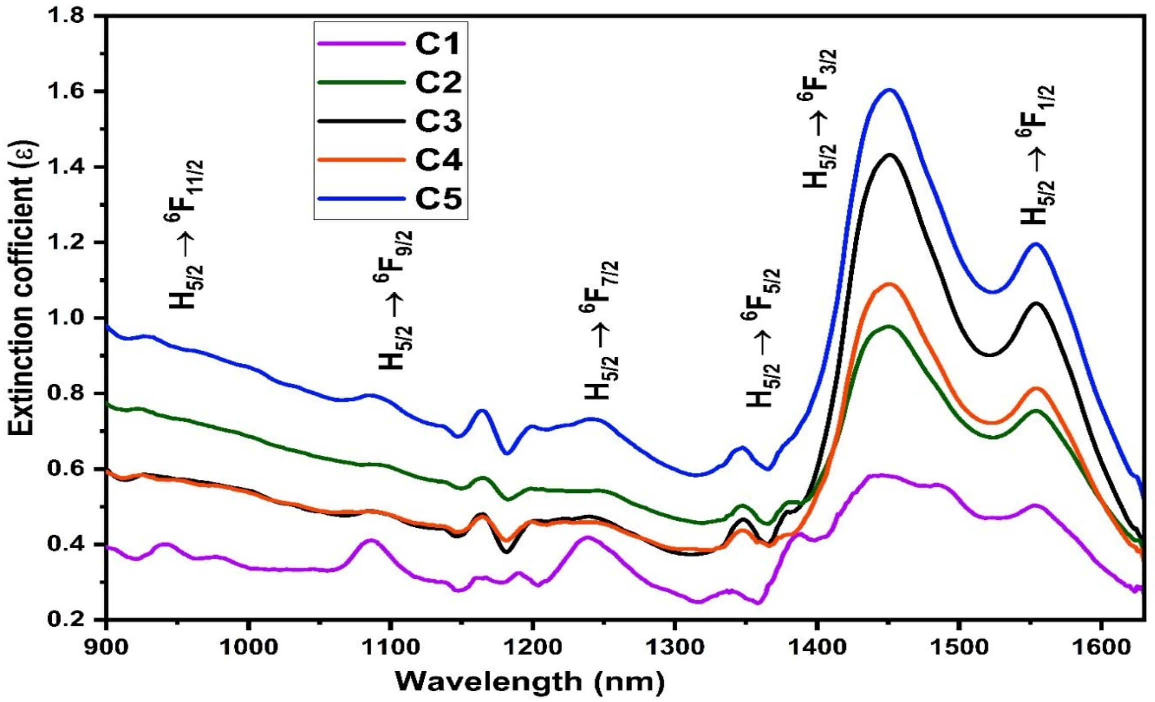

Fig. S8 NIR absorbtion spectra of C1, C2,C3-C6 complexes represening various transitions.

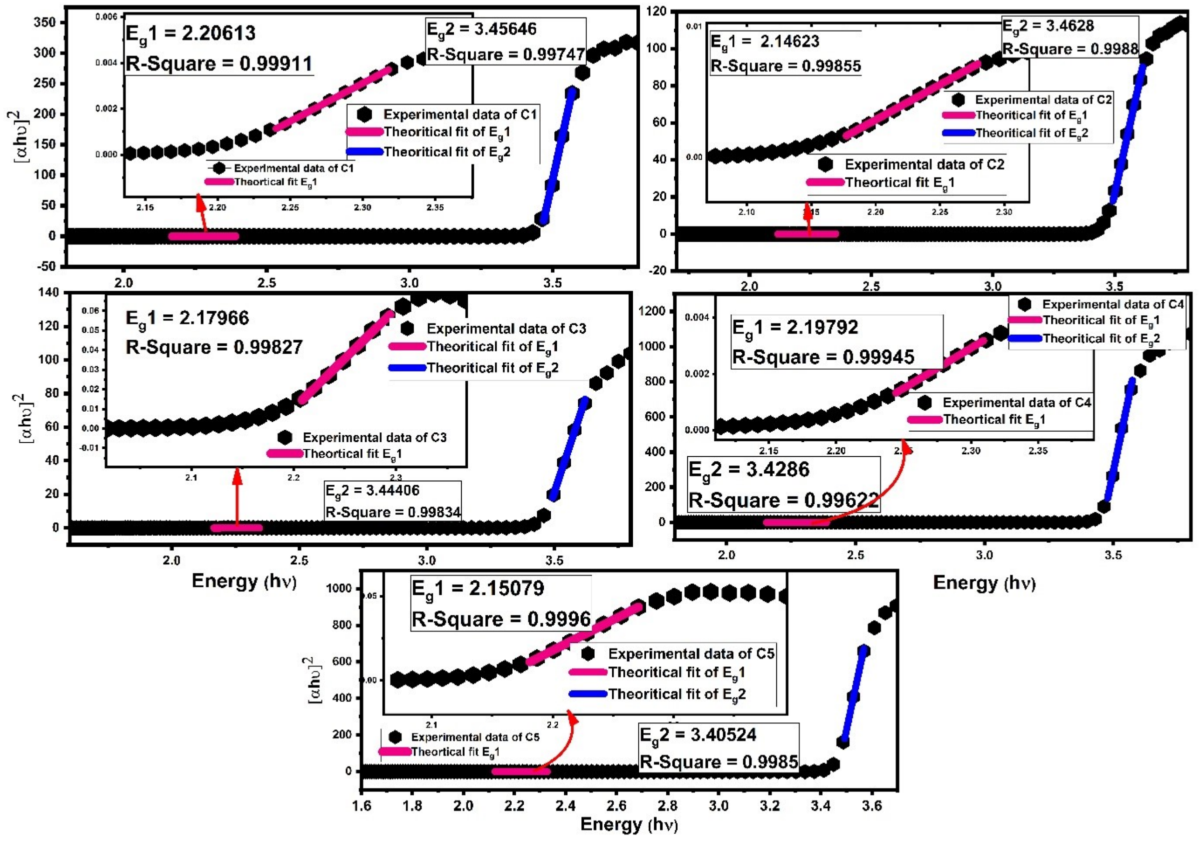

Fig. S9 Linear fitted curve of complexes (C1- C5) using tauc's relation to obtained band gap values (E_g2) in higher energy region whereas in inset of picture shows the zoomed lower energy region with another band gap (E_g1).

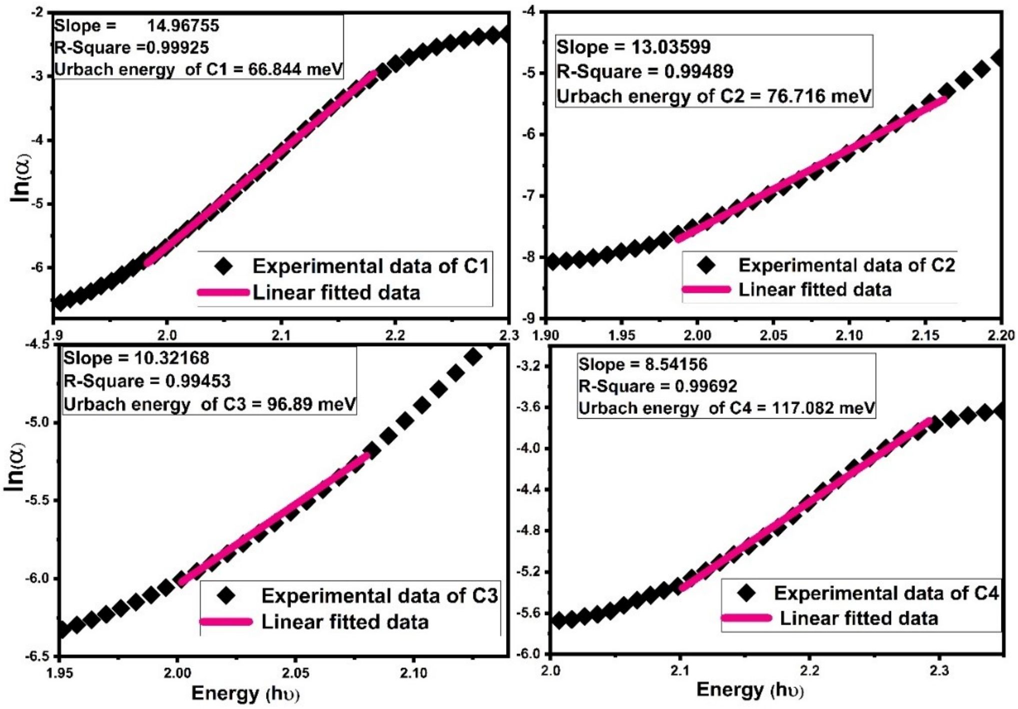

Fig. S10 Fitting of absorption coefficient of C1-C4 complexes using linear fit to obtained Urbach band tail width (U_e)

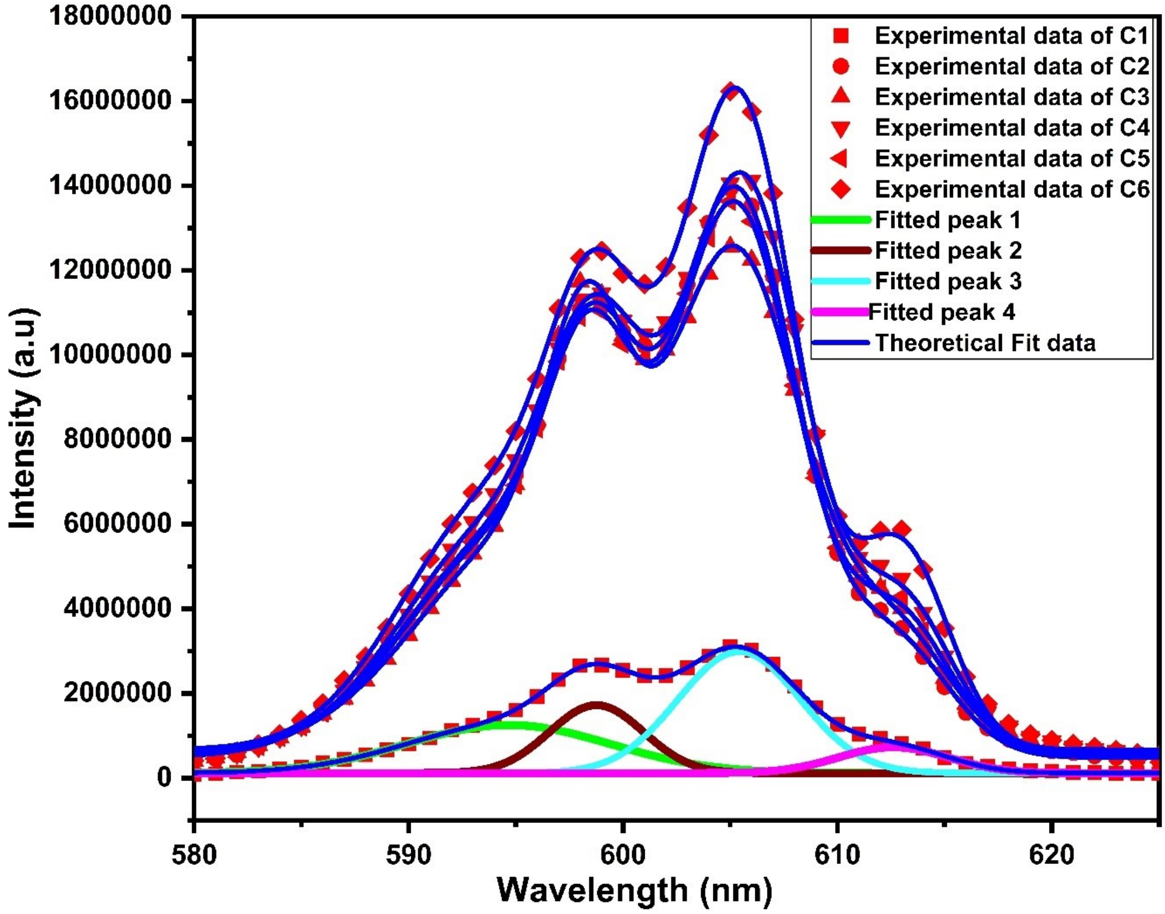

Fig. S11 Deconvolution into four peaks of $^4G_{5/2} \rightarrow {}^6H_{7/2}$ transition by Gaussian Fitting method to show more Starke splitting in solution phase as compared to solid phase emission respectively.

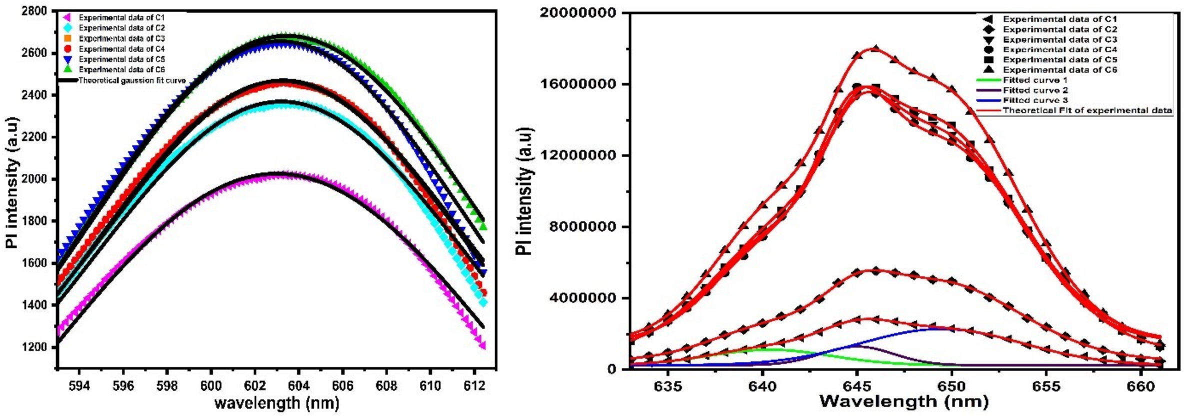

(a) **(b)**

Fig. S12 (a) & S12 (b) To find out FWHM, Gaussian Fitting of $^4G_{5/2} \rightarrow {}^6H_{7/2}$ and $^4G_{5/2} \rightarrow {}^6H_{9/2}$ transitions of solid and solution phase solid phase emission respectively.

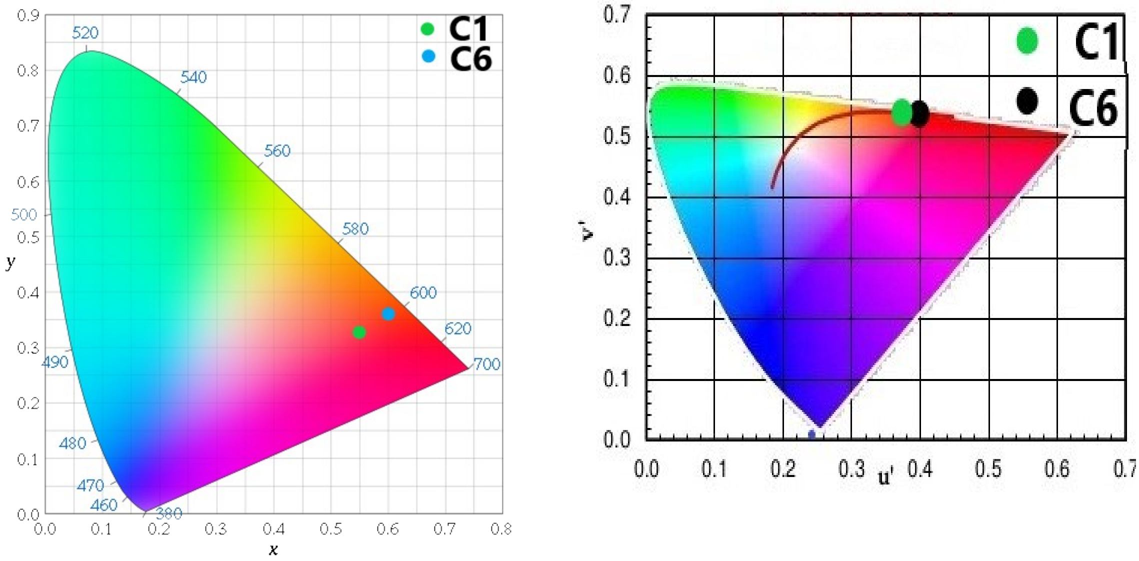

(a) **(b)**

Fig. S13 (a) CIE chromaticity coordinates of complexes C1 and C6 demarcated in radish orange region of colour gamut space. (b) u' and v' coordinates of complexes C1 and C6 in solid state on CCT chart showing their emission in warm radish orange light.

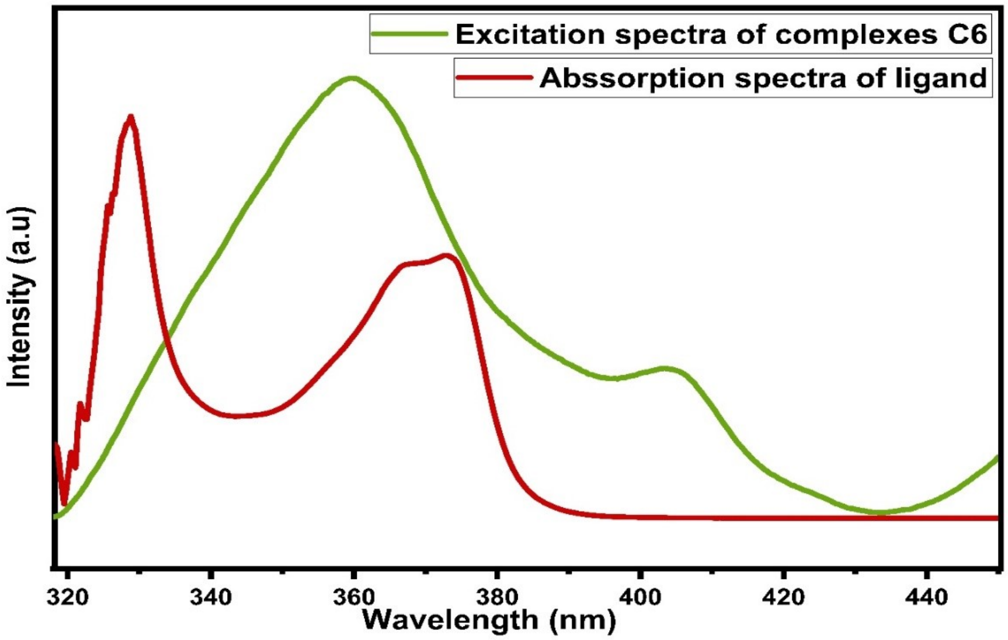

Fig. S14. Overlapping spectra of absorption spectra of L and emission spectra of C1 complex.

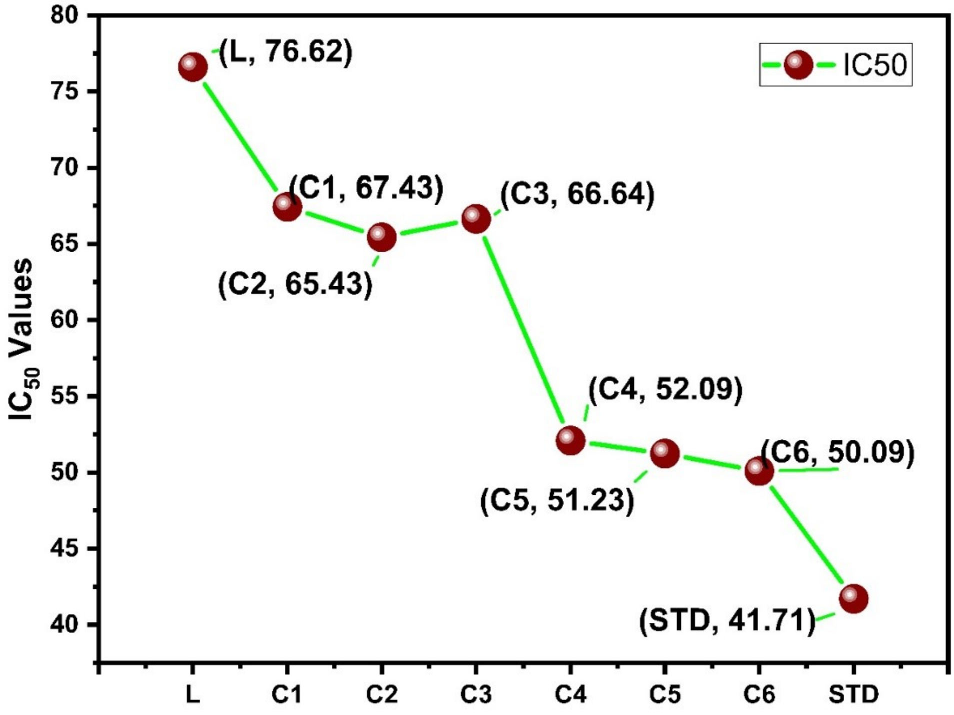

Fig. S15. IC_{50} values of complexes C1-C6 relative to standared drugs.

References:

1 P. Kumari, S. Khatri, M. Kumar, P. Ahlawat, S. P. Khatkar, V. B. Taxak and R. Kumar, *Polyhedron*, 2022, 115847.

2 P. Ahlawat, S. Bhayana, V. Lather, S. Khatri, P. Kumari, M. Kumar, M. S. Shekhawat, V. B. Taxak, S. P. Khatkar and R. Kumar, *Opt. Mater. (Amst).*, 2022, **133**, 112940.
